# LuxT Is a Global Regulator of Low-Cell-Density Behaviors, Including Type III Secretion, Siderophore Production, and Aerolysin Production, in Vibrio harveyi

**DOI:** 10.1128/mbio.03621-21

**Published:** 2022-01-18

**Authors:** Michaela J. Eickhoff, Chenyi Fei, Jian-Ping Cong, Bonnie L. Bassler

**Affiliations:** a Department of Molecular Biology, Princeton University, Princeton, New Jersey, USA; b Lewis-Sigler Institute for Integrative Genomics, Princeton University, Princeton, New Jersey, USA; c Howard Hughes Medical Institute, Chevy Chase, Maryland, USA; National Institute of Child Health and Human Development (NICHD)

**Keywords:** LuxT, gene regulation, quorum sensing, vibrio, virulence

## Abstract

Quorum sensing (QS) is a chemical communication process in which bacteria produce, release, and detect extracellular signaling molecules called autoinducers. Via combined transcriptional and posttranscriptional regulatory mechanisms, QS allows bacteria to collectively alter gene expression on a population-wide scale. Recently, the TetR family transcriptional regulator LuxT was shown to control Vibrio harveyi *qrr*1, encoding the Qrr1 small RNA that functions at the core of the QS regulatory cascade. Here, we use RNA sequencing to reveal that, beyond the control of *qrr*1, LuxT is a global regulator of 414 V. harveyi genes, including those involved in type III secretion, siderophore production, and aerolysin toxin biosynthesis. Importantly, LuxT directly represses *swrZ*, encoding a GntR family transcriptional regulator, and LuxT control of type III secretion, siderophore, and aerolysin genes occurs by two mechanisms, one that is SwrZ dependent and one that is SwrZ independent. All of these target genes specify QS-controlled behaviors that are enacted when V. harveyi is at low cell density. Thus, LuxT and SwrZ function in parallel with QS to drive particular low-cell-density behaviors. Phylogenetic analyses reveal that *luxT* is highly conserved among *Vibrionaceae*, but *swrZ* is less well conserved. In a test case, we find that in Aliivibrio fischeri, LuxT also represses *swrZ*. SwrZ is a repressor of A. fischeri siderophore production genes. Thus, LuxT repression of *swrZ* drives the activation of A. fischeri siderophore gene expression. Our results indicate that LuxT is a major regulator among *Vibrionaceae*, and in the species that also possess *swrZ*, LuxT functions with SwrZ to control gene expression.

## INTRODUCTION

Bacteria have the remarkable ability to rapidly detect and adapt to environmental fluctuations. Often, bacteria employ transcriptional and posttranscriptional regulatory mechanisms to tune gene expression patterns that enhance survival under various conditions (1, 2). Such combined regulatory mechanisms are used in a process called quorum sensing (QS) to monitor and react to changes in the cell density and the species composition of the vicinal community. QS involves the production, release, accumulation, and group-wide detection of signaling molecules called autoinducers (AIs). QS fosters the synchronous execution of collective behaviors, typically ones that are unproductive for an individual bacterium to carry out alone but that become effective when enacted by the group, e.g., bioluminescence, virulence factor production, and biofilm formation (3, 4).

Vibrio harveyi, the focus of the current work, is a model QS bacterium that uses three AIs that act in parallel to control bioluminescence, type III secretion, siderophore production, and hundreds of other traits (5–8). Each AI is detected by a cognate membrane-bound receptor. At low cell density (LCD), AI concentrations are low, and the three unliganded QS receptors act as kinases that drive the phosphorylation of the response regulator LuxO. Phosphorylated LuxO (LuxO-P) activates the transcription of genes encoding five small regulatory RNAs (sRNAs) called the Qrr sRNAs that function posttranscriptionally to activate and repress the translation of the master QS regulators AphA and LuxR, respectively (9–12). Thus, at LCD, AphA is made, and LuxR is not. AphA is responsible for executing the LCD QS program (see Fig. S1A in the supplemental material). At LCD, the Qrr sRNAs also directly control 16 other target mRNAs, and they operate as feedback regulators within the QS signaling pathway (13–15). At high cell density (HCD), accumulated AIs bind to their cognate QS receptors. In the liganded state, the QS receptors act as phosphatases, and phosphate is stripped from LuxO, which inactivates it (16, 17). Thus, at HCD, Qrr sRNA production terminates, AphA is not made, and, in contrast, LuxR is produced (8, 10, 12). LuxR drives the HCD QS regulon (Fig. S1B).

10.1128/mbio.03621-21.1 FIG S1 Simplified V. harveyi QS pathway. LCD (A) and HCD (B) V. harveyi QS circuits are shown. Circles, triangles, and squares represent AIs. See the introduction in the main text for details. Download FIG S1, PDF file, 0.02 MB. Copyright © 2022 Eickhoff et al. 2022 Eickhoff et al. https://creativecommons.org/licenses/by/4.0/ This content is distributed under the terms of the Creative Commons Attribution 4.0 International license.

The five V. harveyi Qrr sRNAs (Qrr1–5) possess high sequence identity, and Qrr2–5 regulate an identical set of mRNA targets (10, 13). Qrr1 is the outlier. Because it lacks 9 nucleotides that are present in Qrr2–5, Qrr1 fails to regulate *aphA* and two additional mRNA targets that are controlled by Qrr2–5 (10, 11, 13). The genes encoding the Qrr sRNAs differ in their LCD expression levels: *qrr*4 is the most highly transcribed of the set, whereas only low-level transcription of *qrr*1 and *qrr*5 occurs (10). Recently, we discovered that a transcription factor called LuxT represses the expression of *qrr*1. LuxT does not regulate *qrr*2–5. Thus, the exclusive involvement of LuxT in *qrr*1 regulation provides a mechanism enabling Qrr1 to uniquely control downstream targets (18). Indeed, via the repression of *qrr*1, LuxT indirectly controls the translation of Qrr1 target mRNAs, including those encoding a secreted protease, an aerolysin toxin, a chitin deacetylase, and a component involved in capsule polysaccharide secretion. LuxT also activates the transcription of this same set of genes by a Qrr1-independent mechanism (18). LuxT repression of *qrr*1 does not significantly alter LuxR translation, and as mentioned above, Qrr1 does not regulate AphA (11, 18). These findings indicate that LuxT repression of *qrr*1 tunes the expression of select Qrr1-controlled mRNAs without altering the entire QS regulon.

To date, *qrr*1 is the only gene identified to be directly controlled by LuxT (18, 19). However, LuxT has been linked to the regulation of additional phenotypes in V. harveyi and other *Vibrionaceae*, including bioluminescence, siderophore production, virulence factor production, and motility (18, 20–23). These findings, together with our initial knowledge that LuxT can regulate the transcription of target genes independently of Qrr1 in V. harveyi (18), inspired us to investigate the role of LuxT beyond its control of *qrr*1. Here, we use RNA sequencing (RNA-Seq) to identify the LuxT regulon, revealing LuxT to be a global regulator of ∼414 genes in V. harveyi. We find that LuxT directly represses the V. harveyi *swrZ* gene encoding a GntR family transcriptional regulator. We use genetic and molecular analyses to show that in V. harveyi, LuxT activates genes required for type III secretion, siderophore production, and aerolysin toxin production, and activation occurs by two mechanisms. One mechanism depends on SwrZ: LuxT represses *swrZ*, and SwrZ represses target gene expression. The second mechanism is SwrZ independent. Finally, the LuxT-controlled traits identified here are also QS controlled and are enacted primarily at LCD. Therefore, LuxT functions in parallel with QS to establish V. harveyi LCD behaviors. Finally, we analyze *luxT* and *swrZ* conservation among *Vibrionaceae* bacteria, and we demonstrate that via *swrZ* repression, LuxT also activates genes required for siderophore production in Aliivibrio fischeri.

## RESULTS

### LuxT is a global regulator that directly represses *swrZ* transcription.

**FIG 1:**
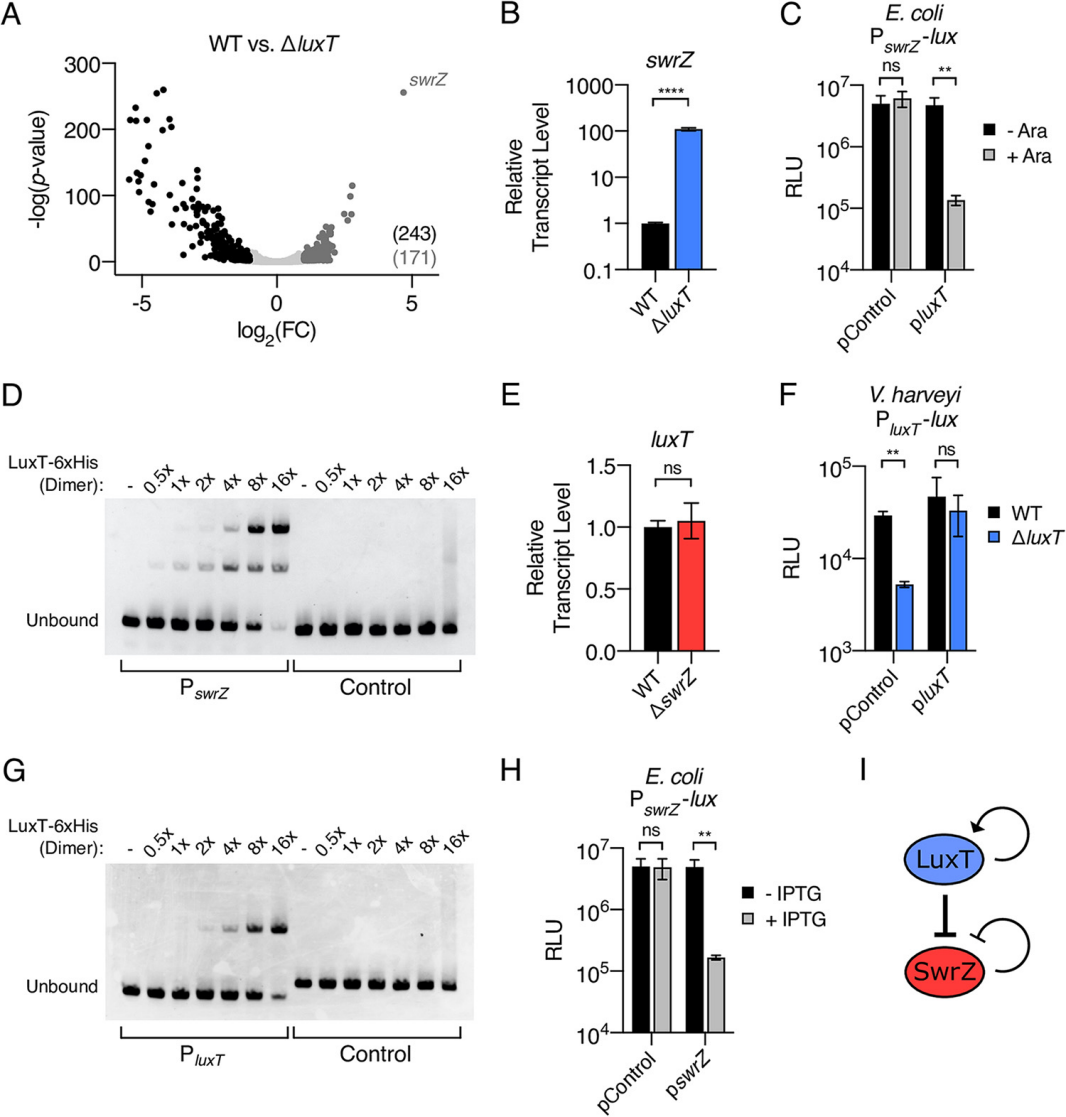
LuxT is a global transcriptional regulator in V. harveyi that directly represses *swrZ*. (A) Volcano plot depicting RNA-Seq data comparing the transcriptome of Δ*luxT* V. harveyi to that of WT V. harveyi. Each data point represents one V. harveyi gene. FC, fold change. A total of 243 genes were significantly activated (black) and 171 genes were significantly repressed (dark gray) by LuxT. (B) qRT-PCR measurements of *swrZ* transcript levels in WT (black) and Δ*luxT* (blue) V. harveyi. RNA was isolated from strains grown in LM medium to an OD_600_ of 0.1. (C) Activity of a plasmid-borne P*_swrZ_-lux* transcriptional reporter in recombinant E. coli. pControl denotes that the second introduced plasmid is the empty parent vector, and p*luxT* designates that the second vector harbors arabinose-inducible *luxT*. Strains were grown in LB to an OD_600_ of 1 in the absence (black) or presence (gray) of 0.01% arabinose. (D) EMSA showing binding of LuxT-6×His to a 113-bp DNA fragment containing the *swrZ* promoter (left) and a 107-bp control fragment containing the V. harveyi *luxC* promoter (right). Previously, it was shown that LuxT does not bind the *luxC* promoter (18). The DNA probe (20 nM) was incubated with the indicated relative concentrations of the LuxT-6×His dimer. - indicates no protein, 1× indicates 20 nM, and 16× indicates 320 nM. (E) qRT-PCR measurements of *luxT* transcript levels in WT (black) and Δ*swrZ* (red) V. harveyi. Growth conditions are the same as those described above for panel B. (F) Activity of a plasmid-borne P*_luxT_-lux* transcriptional reporter in Δ*luxA* (black) and Δ*luxA* Δ*luxT* (blue) V. harveyi strains. pControl and p*luxT* denote the empty parent vector and a vector harboring IPTG-inducible *luxT*, respectively. Strains were grown in LM medium supplemented with 0.5 mM IPTG to an OD_600_ of 1. (G) Same as for panel D, for a 99-bp DNA fragment containing the *luxT* promoter (left) and a 107-bp control fragment containing the V. harveyi *luxC* promoter (right). (H) Activity of a plasmid-borne P*_swrZ_-lux* transcriptional reporter in recombinant E. coli. pControl denotes that the second introduced plasmid is the empty parent vector, and p*swrZ* designates that the second vector harbors IPTG-inducible *swrZ*. Strains were grown in LB to an OD_600_ of 1 in the absence (black) or presence (gray) of 0.5 mM IPTG. (I) Model for the LuxT/SwrZ regulatory circuit. In panels C, F, and H, RLU denotes relative light units. In panels B, C, E, F, and H, error bars represent standard deviations of the means from 3 biological replicates. Unpaired two-tailed *t* tests with Welch’s correction were performed comparing the indicated conditions. ns, not significant (*P* ≥ 0.05); **, *P* < 0.01; ****, *P* < 0.0001.

To define the set of genes regulated by LuxT, we used RNA-Seq to compare the transcriptomes of wild-type (WT) and Δ*luxT* V. harveyi. We considered transcripts with changes in expression of 2-fold or higher (*P < *0.01) to be LuxT regulated, revealing a total of 414 genes: 243 activated and 171 repressed (Fig. 1A; see also Data Set S1 in the supplemental material). One gene, *swrZ* (*VIBHAR_RS03920*), stood out due to its dramatic repression by LuxT. *swrZ* was previously shown to be repressed by the LuxT homolog SwrT in Vibrio parahaemolyticus (22). Specifically, SwrT repressed the transcription of *swrZ*, and SwrZ repressed the *laf* genes encoding the lateral flagellar machinery required for V. parahaemolyticus swarming locomotion. Thus, through this cascade, repression of a repressor, SwrT activates V. parahaemolyticus swarming (22). While V. harveyi carries *laf* genes, their expression levels are extremely low, and V. harveyi swarming has not been documented (24). The V. harveyi *laf* genes were not revealed by RNA-Seq to be LuxT regulated. Nonetheless, our data demonstrate that the regulatory arrangement in which LuxT represses *swrZ* exists in V. harveyi: quantitative real-time PCR (qRT-PCR) verified that LuxT represses *swrZ* expression by ∼100-fold (Fig. 1B).

10.1128/mbio.03621-21.10 DATA SET S1 The V. harveyi LuxT regulon. Genes activated (sheet 1) and repressed (sheet 2) by LuxT in V. harveyi are shown. FC, fold change in gene expression in the Δ*luxT* strain compared to the WT. Download Data Set S1, XLSX file, 0.1 MB. Copyright © 2022 Eickhoff et al. 2022 Eickhoff et al. https://creativecommons.org/licenses/by/4.0/ This content is distributed under the terms of the Creative Commons Attribution 4.0 International license.

To determine if LuxT repression of *swrZ* is direct, we used recombinant Escherichia coli to isolate LuxT and the *swrZ* promoter from other V. harveyi regulatory components. Two plasmids were introduced into E. coli. One plasmid harbored a P*_swrZ_-lux* transcriptional reporter, and the second plasmid carried arabinose-inducible *luxT*. The induction of *luxT* expression drove the repression of P*_swrZ_-lux* in the E. coli strain carrying the p*luxT* vector, whereas no repression occurred in E. coli carrying the empty control vector (Fig. 1C). We conclude that LuxT directly represses *swrZ*. Consistent with this supposition, purified LuxT-6×His protein bound to the *swrZ* promoter in an *in vitro* electrophoretic mobility shift assay (EMSA), whereas LuxT-6×His did not bind to control DNA (Fig. 1D). The laddering present in the P*_swrZ_* EMSA may indicate that multiple LuxT-binding sites exist in the *swrZ* promoter and/or that LuxT oligomerizes when binding the *swrZ* promoter (Fig. 1D).

### Both *luxT* and *swrZ* are subject to feedback regulation, and neither *luxT* nor *swrZ* is controlled by QS.

To further explore the connections between LuxT and SwrZ in V. harveyi, we assessed whether feedback regulatory loops exist. First, regarding SwrZ regulation of *luxT*, we find no evidence for SwrZ-mediated feedback onto *luxT* because *luxT* transcript levels were similar in WT and Δ*swrZ* V. harveyi (Fig. 1E); moreover, the overexpression of *swrZ* in WT V. harveyi did not alter *luxT* transcription as measured by qRT-PCR (Fig. S2). qRT-PCR validated that *swrZ* was indeed overexpressed from the p*swrZ* plasmid (Fig. S2). Second, we investigated autoregulation of *luxT*. The activity of a P*_luxT_-lux* reporter was measured in Δ*luxA* and Δ*luxA* Δ*luxT* V. harveyi strains. V. harveyi is naturally bioluminescent, and *luxA* encodes a luciferase subunit. Thus, deletion of *luxA* was required to ensure that all light produced by the test strains came from the P*_luxT_*-*lux* reporter. Figure 1F shows that in strains harboring an empty control vector, P*_luxT_-lux* expression level is 6-fold higher in the Δ*luxA* V. harveyi strain than in the Δ*luxA* Δ*luxT* strain. The introduction of a vector expressing *luxT* restored light production (Fig. 1F). Thus, LuxT activates its own transcription. Unfortunately, autoregulation of *luxT* could not be tested in recombinant E. coli because the P*_luxT_-lux* reporter was not expressed to any detectable level. However, in an *in vitro* EMSA, LuxT-6×His bound the *luxT* promoter, providing evidence for a direct autoregulatory role (Fig. 1G). Finally, we investigated direct autoregulation of *swrZ*. In recombinant E. coli, the induction of *swrZ* expression repressed a P*_swrZ_-lux* reporter 30-fold, indicating direct feedback repression (Fig. 1H). We conclude that LuxT and SwrZ can be placed into a regulatory pathway in which LuxT directly represses *swrZ*. Positive and negative feedback loops exist for LuxT and SwrZ, respectively (Fig. 1I). Below, we speculate on the implications of this regulatory arrangement.

10.1128/mbio.03621-21.2 FIG S2 SwrZ does not regulate *luxT*. Shown are qRT-PCR measurements of *luxT* and *swrZ* transcript levels in WT V. harveyi harboring the following plasmids: pControl (black) denotes the empty parent vector, and p*swrZ* (red) denotes a vector carrying IPTG-inducible *swrZ*. RNA was isolated from strains grown in LM medium containing 0.5 mM IPTG to an OD_600_ of 1. Unpaired two-tailed *t* tests with Welch’s correction were performed comparing the pControl and p*swrZ* conditions for each gene. ns, not significant (*P* ≥ 0.05); ***, *P* < 0.001. Download FIG S2, PDF file, 0.02 MB. Copyright © 2022 Eickhoff et al. 2022 Eickhoff et al. https://creativecommons.org/licenses/by/4.0/ This content is distributed under the terms of the Creative Commons Attribution 4.0 International license.

Analysis of the LuxT regulon revealed that a subset of LuxT-controlled genes is also regulated by QS (8) (Data Set S1). One possible explanation for this finding is that regulatory interconnections exist between LuxT and QS. We know that LuxT does not regulate *aphA* or *luxR*, so LuxT cannot reside upstream of these two components in the QS cascade (18). Alternatively, QS could control *luxT* and/or *swrZ* expression. To test this possibility, we measured *luxT* and *swrZ* expression in WT, Δ*luxO*, and *luxO* D61E V. harveyi strains. The logic is as follows. The WT strain undergoes the normal LCD-to-HCD QS transition. The Δ*luxO* strain exhibits “HCD-locked” behavior because in the absence of LuxO activity, no Qrr sRNAs are produced (9, 10) (Fig. S1B). *luxO* D61E encodes a LuxO-P mimetic, so the strain harboring this mutation exhibits “LCD-locked” behavior in which the Qrr sRNAs are constitutively produced (10, 17) (Fig. S1A). There were no significant differences in *luxT* or *swrZ* expression in these strains (Fig. S3A). Consistent with this finding, no dramatic changes in *luxT* expression occurred over growth as would be expected for a QS-regulated gene (Fig. S3B). As expected from our above-described findings (Fig. 1B), *swrZ* transcript levels were >100-fold higher in the Δ*luxT* strain than in WT V. harveyi, but again, cell-density-dependent changes in *swrZ* expression did not occur in either strain, arguing against QS control (Fig. S3C). We conclude that the expression of *luxT* and *swrZ* is not QS regulated. Thus, we infer that QS and LuxT/SwrZ function independently and in parallel in the regulation of a subset of genes in each of their regulons.

10.1128/mbio.03621-21.3 FIG S3 QS does not regulate *luxT* or *swrZ* in V. harveyi. (A) qRT-PCR measurements of *luxT* and *swrZ* transcript levels in WT (black), Δ*luxO* (green), and *luxO* D61E (orange) V. harveyi strains. RNA was isolated from strains grown in AB medium to an OD_600_ of 1. (B) qRT-PCR measurements of *luxT* transcript levels over growth. RNA was isolated from WT V. harveyi grown in LM medium to different cell densities, as indicated by the OD_600_ values on the *x* axis. (C) Same as for panel B, except *swrZ* transcript levels were measured in WT (black) and Δ*luxT* (blue) V. harveyi strains. In all panels, error bars represent standard deviations of the means from 3 biological replicates. Standard deviations that are smaller than the symbols are not shown. In panel A, different letters indicate significant differences between strains (*P < *0.05 by two-way analysis of variation [ANOVA] followed by Tukey’s multiple-comparison test). Download FIG S3, PDF file, 0.1 MB. Copyright © 2022 Eickhoff et al. 2022 Eickhoff et al. https://creativecommons.org/licenses/by/4.0/ This content is distributed under the terms of the Creative Commons Attribution 4.0 International license.

### LuxT activates the expression of type III secretion system genes via repression of *swrZ*.

**FIG 2:**
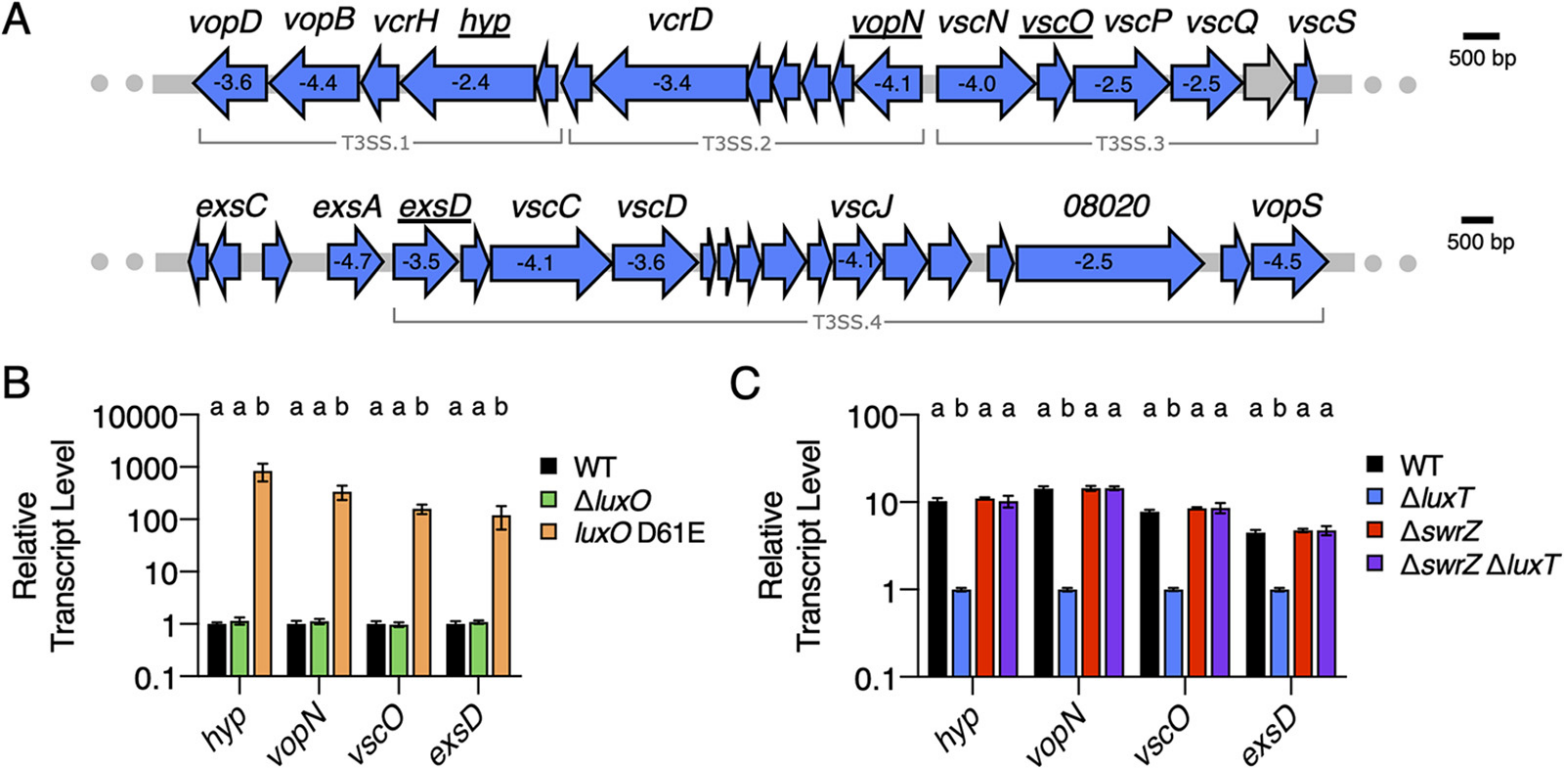
QS and LuxT/SwrZ regulate V. harveyi T3SS gene expression. (A) Schematic of T3SS gene organization in V. harveyi. There are four major T3SS operons, as labeled. All genes shown in blue were identified by RNA-Seq as members of the LuxT regulon. The numbers shown within select genes designate the fold change differences in transcript levels between WT and Δ*luxT* V. harveyi, as measured by RNA-Seq (see Data Set S1 in the supplemental material). The expression of the underlined genes was measured in panels B and C. (B) qRT-PCR measurements of mRNA levels of the indicated genes in WT (black), Δ*luxO* (green), and *luxO* D61E (orange) V. harveyi strains. RNA was isolated from strains grown in AB medium to an OD_600_ of 1. (C) qRT-PCR measurements of mRNA levels of the indicated genes in WT (black), Δ*luxT* (blue), Δ*swrZ* (red), and Δ*swrZ* Δ*luxT* (purple) V. harveyi strains. RNA was isolated from strains grown in LM medium to an OD_600_ of 0.1. In panels B and C, error bars represent standard deviations of the means from 3 biological replicates. Different letters indicate significant differences between strains (*P < *0.05 by two-way analysis of variation [ANOVA] followed by Tukey’s multiple-comparison test).

As mentioned above, LuxT regulates ∼413 genes in addition to *swrZ* (Fig. 1A and Data Set S1). To gain insight into the LuxT regulon, as examples, we characterize the mechanisms by which LuxT controls genes specifying three V. harveyi functions: type III secretion, siderophore production, and aerolysin production. We begin with type III secretion genes. Type III secretion systems (T3SSs) are syringe-like molecular machines that ferry toxic effector proteins across bacterial inner and outer membranes and into target eukaryotic cells (25–27). In V. harveyi and other vibrios, type III secretion is crucial for virulence (28–30). The structural proteins comprising the V. harveyi T3SS are encoded in four operons (T3SS.1, T3SS.2, T3SS.3, and T3SS.4) (Fig. 2A) (5, 8, 31). Type III secretion is QS regulated in V. harveyi (5). Specifically, T3SS genes are repressed by both AphA and LuxR. The consequence is that T3SS gene expression peaks at low- to mid-cell density when AphA levels have decreased and LuxR levels have not risen (8, 12). QS regulation of type III secretion occurs indirectly through ExsA, the master transcriptional activator of T3SS genes (Fig. 2A). Both AphA and LuxR directly repress *exsA* (8, 31).

The genes in all four T3SS operons and *exsA* were revealed by RNA-Seq to be activated by LuxT (Fig. 2A and Data Set S1). To understand the dual control of T3SS genes by QS and LuxT, we engineered a set of strains and developed a companion strategy to probe the regulatory mechanisms. First, we verified LuxT involvement from the RNA-Seq analysis, and we determined if *qrr*1 is required for LuxT control of T3SS genes. Using qRT-PCR, we quantified the transcript levels of four T3SS genes, *hyp*, *vopN*, *vscO*, and *exsD*, representing the four T3SS operons (underlined in Fig. 2A). Expression levels were compared between WT and Δ*luxT* V. harveyi and between Δ*qrr*1 and Δ*qrr*1 Δ*luxT* V. harveyi. Expression levels were ∼10-, 14-, 8-, and 4-fold lower, respectively, in the Δ*luxT* strain than in the WT, confirming that LuxT is an activator of T3SS genes (Fig. S4A). Results nearly identical to those shown in Fig. S4A were obtained for the Δ*qrr*1 and Δ*qrr*1 Δ*luxT* strains, indicating that LuxT activates T3SS genes independently of Qrr1 (Fig. S4B).

10.1128/mbio.03621-21.4 FIG S4 LuxT activates T3SS, siderophore, and aerolysin genes by Qrr1-independent mechanisms in V. harveyi. Shown are qRT-PCR measurements of transcript levels of the indicated genes in WT (black) and Δ*luxT* (blue) V. harveyi strains (A, C, and E) or in Δ*qrr*1 (black) and Δ*qrr*1 Δ*luxT* (blue) V. harveyi strains (B, D, and F). RNA was isolated from strains grown in LM medium to an OD_600_ of 0.1. In all panels, error bars represent standard deviations of the means from 3 biological replicates. Unpaired two-tailed *t* tests with Welch’s correction were performed comparing two conditions, as indicated. *, *P* < 0.05; **, *P* < 0.01; ***, *P* < 0.001; ****, *P* < 0.0001. Download FIG S4, PDF file, 0.1 MB. Copyright © 2022 Eickhoff et al. 2022 Eickhoff et al. https://creativecommons.org/licenses/by/4.0/ This content is distributed under the terms of the Creative Commons Attribution 4.0 International license.

To verify the mechanism by which QS regulates T3SS genes, we measured the transcript levels of the four representative genes in WT, Δ*luxO* (HCD-locked), and *luxO* D61E (LCD-locked) V. harveyi strains. The expression levels of *hyp*, *vopN*, *vscO*, and *exsD* were >100-fold higher in the *luxO* D61E strain than in the WT and Δ*luxO* strains at HCD (Fig. 2B). Consistent with previously published results, high-level expression of T3SS genes occurs at LCD due to LuxR-mediated repression of them at HCD (5, 8). Specifically, compared to the WT, a Δ*luxR* V. harveyi strain exhibited >100-fold-higher expression levels of the four genes (Fig. S5A). AphA is known to repress T3SS.1 and T3SS.4 in V. harveyi (8). The expression levels of *hyp* (T3SS.1) and *exsD* (T3SS.4) were ∼1.6-fold higher in the *luxO* D61E Δ*aphA* strain than in the *luxO* D61E strain; however, the differences were not statistically significant (Fig. S5B). We presume that we did not observe a larger role for AphA here because of differences in our experimental growth conditions compared to those used previously and the use of qRT-PCR compared to microarrays (8).

10.1128/mbio.03621-21.5 FIG S5 V. harveyi T3SS genes are repressed at HCD by LuxR. (A) qRT-PCR measurements of transcript levels of the indicated T3SS genes in WT (black) and Δ*luxR* (gray) V. harveyi strains. RNA was isolated from strains grown in AB medium to an OD_600_ of 1. (B) Same as for panel A, except transcript levels were measured in *luxO* D61E (black) and *luxO* D61E Δ*aphA* (gray) V. harveyi strains. In both panels, error bars represent standard deviations of the means from 3 biological replicates. Unpaired two-tailed *t* tests with Welch’s correction were performed comparing two conditions, as indicated. ns, not significant (*P* ≥ 0.05); *, *P* < 0.05; **, *P* < 0.01. Download FIG S5, PDF file, 0.04 MB. Copyright © 2022 Eickhoff et al. 2022 Eickhoff et al. https://creativecommons.org/licenses/by/4.0/ This content is distributed under the terms of the Creative Commons Attribution 4.0 International license.

To assess the mechanism by which LuxT controls T3SS gene expression, we first determined whether the LuxT-mediated activation of T3SS genes requires SwrZ by measuring the transcript levels of the four representative T3SS genes in WT, Δ*luxT*, Δ*swrZ*, and Δ*swrZ* Δ*luxT* V. harveyi strains at LCD. Comparison of expression levels between the WT and Δ*luxT* strains validates LuxT activation of the genes. Comparison of expression levels between the Δ*swrZ* and Δ*swrZ* Δ*luxT* strains tests whether LuxT activation of genes requires SwrZ. As described above (Fig. S4A), the expression levels of *hyp*, *vopN*, *vscO*, and *exsD* were lower in the Δ*luxT* strain than in the WT (Fig. 2C). However, the elimination of *luxT* did not alter transcript levels in the Δ*swrZ* background (Fig. 2C). Thus, LuxT activates the expression of all four T3SS operons via a SwrZ-dependent mechanism. Most likely, LuxT represses *swrZ*, and SwrZ represses T3SS genes. We test this assumption below.

### LuxT regulates *exsA* by SwrZ-dependent and SwrZ-independent mechanisms to activate type III secretion.

**FIG 3:**
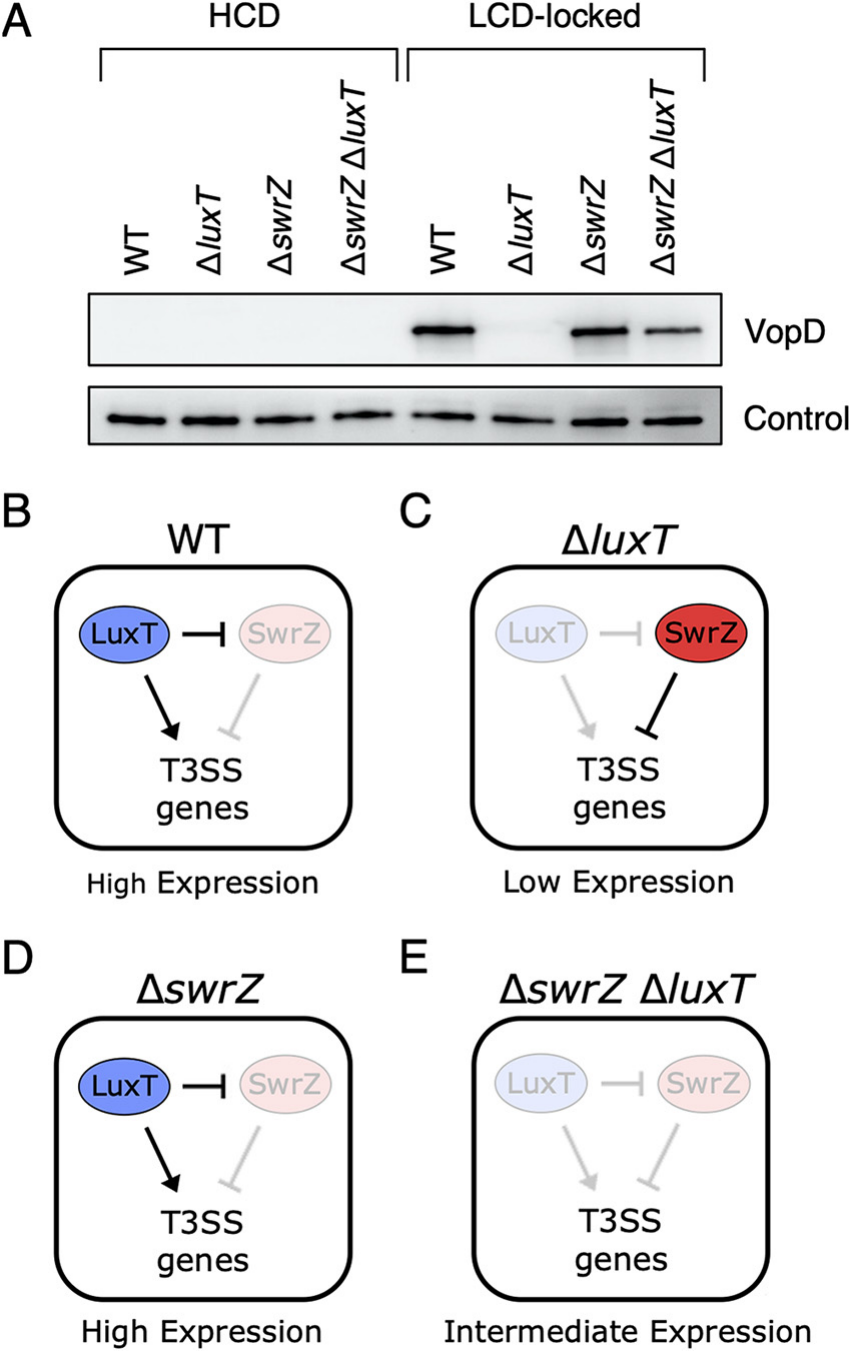
LuxT activates V. harveyi type III secretion gene expression by two mechanisms. (A) Western blot of cytoplasmic VopD levels in the indicated V. harveyi strains at HCD. “LCD-locked” denotes that the parent strain for the samples in the right four lanes harbors the *luxO* D61E mutation. Detection of the LuxS protein serves as the loading control, as previously described (5). (B to E) Schematics depicting the outcomes for LuxT regulation of T3SS genes by SwrZ-dependent and SwrZ-independent mechanisms for WT (B), Δ*luxT* (C), Δ*swrZ* (D), and Δ*swrZ* Δ*luxT* (E) scenarios. See the text for details.

To quantify the contributions of QS and LuxT/SwrZ to the regulation of type III secretion, we used Western blot assessment of VopD, a T3SS.1 protein that accumulates in and is secreted from the cytoplasm following the activation of T3SS gene expression (5). We measured VopD levels in WT, Δ*luxT*, Δ*swrZ*, and Δ*swrZ* Δ*luxT* V. harveyi strains at HCD and in the LCD-locked (*luxO* D61E) background strain. No VopD was detected in the strains at HCD, consistent with LuxR repression of T3SS genes (Fig. 3A, first four lanes) (5, 31). In contrast, VopD was made in the *luxO* D61E strain, and deletion of *luxT* in the *luxO* D61E strain reduced VopD levels to below our detection limit (Fig. 3A, fifth and sixth lanes). These data mirror our qRT-PCR results from Fig. 2C and confirm the role of LuxT as an activator of type III secretion. To assess whether *swrZ* is required for LuxT activation of type III secretion, VopD levels were measured in *luxO* D61E Δ*swrZ* and *luxO* D61E Δ*swrZ* Δ*luxT* strains. Lower levels of VopD were present in the *luxO* D61E Δ*swrZ* Δ*luxT* strain than in the *luxO* D61E Δ*swrZ* strain (Fig. 3A, rightmost two lanes). These results differ from the qRT-PCR results in Fig. 2C, likely because of the different growth conditions required for the qRT-PCR and the VopD Western blot analyses. We summarize the data in Fig. 3A as follows: deletion of *luxT* reduces VopD production in the presence and absence of *swrZ*; however, a more dramatic reduction occurs when *swrZ* is present. To explain these data, we developed and tested the following model for LuxT/SwrZ regulation of T3SS genes.

We propose that LuxT activates T3SS genes by two mechanisms: one mechanism is SwrZ dependent, and one mechanism is SwrZ independent. This model is depicted in Fig. 3B to E and is used to explain the data for the LCD-locked strains in the Western blot shown in Fig. 3A (rightmost four lanes). In WT V. harveyi (Fig. 3B), LuxT is present. It represses *swrZ*, and it also activates T3SS genes by a SwrZ-independent mechanism. The consequence is LuxT activation of T3SS genes. Therefore, high-level VopD production occurs (Fig. 3A, fifth lane). In Δ*luxT* V. harveyi (Fig. 3C), *swrZ* is derepressed. The consequence is that SwrZ is produced and available to repress T3SS genes. Therefore, no VopD is produced (Fig. 3A, sixth lane). In the Δ*swrZ* strain (Fig. 3D), the situation is essentially identical to that of the WT in which *swrZ* is repressed by LuxT, but in this case, there simply is no *swrZ*. The consequence is LuxT activation of T3SS genes. Therefore, high-level VopD production occurs (Fig. 3A, seventh lane). Finally, in the case of the Δ*swrZ* Δ*luxT* double mutant (Fig. 3E), there is no repression of T3SS genes by SwrZ, and there is no activation by LuxT. Thus, basal-level expression of T3SS genes ensues, and an intermediate amount of VopD is made (Fig. 3A, eighth lane). Key to this model is that VopD levels in the LCD-locked Δ*swrZ* and Δ*swrZ* Δ*luxT* strains are not identical because of the SwrZ-independent mechanism by which LuxT activates T3SS genes (Fig. 3D and E).

**FIG 4:**
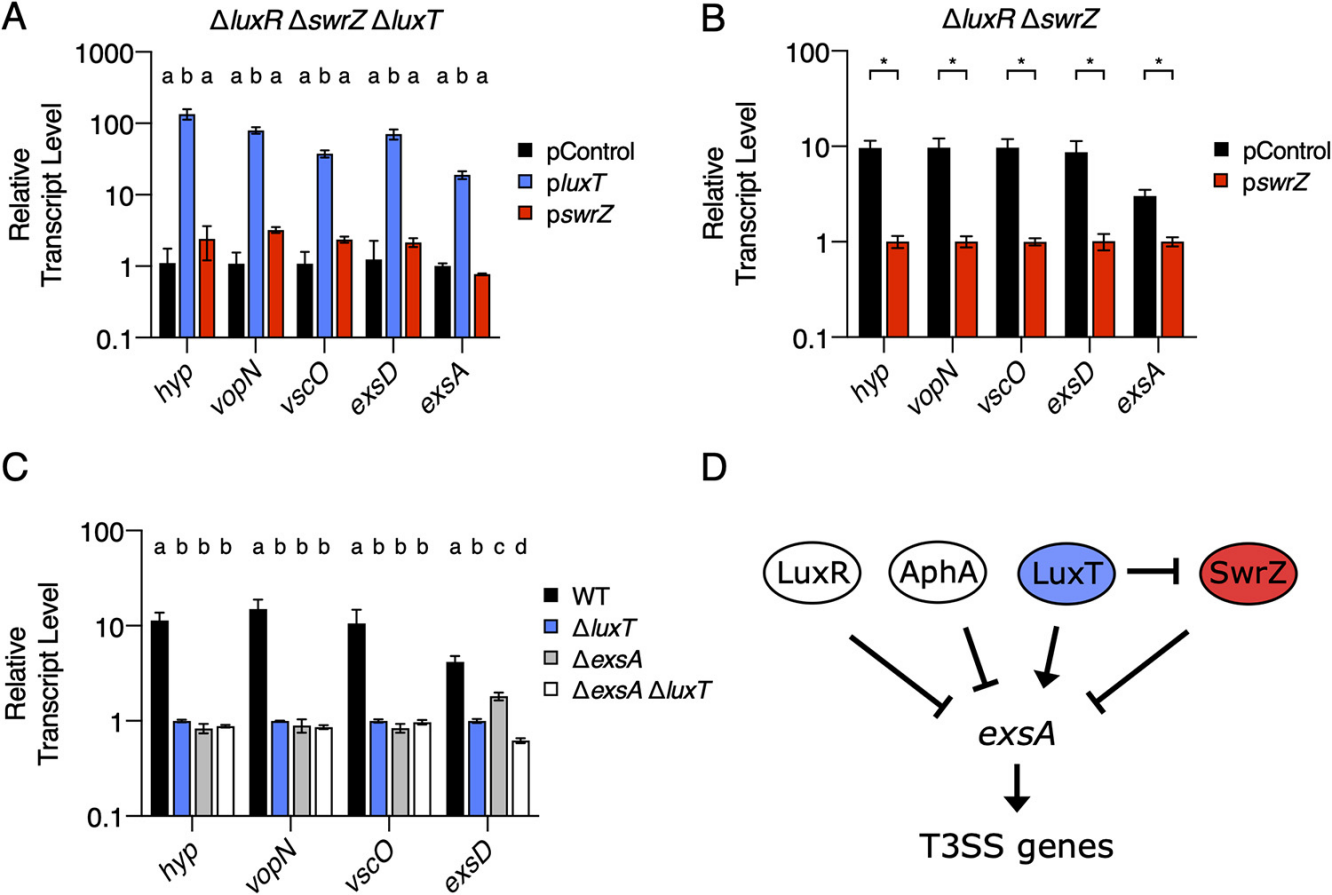
LuxT activates V. harveyi *exsA* and, in turn, T3SS genes by SwrZ-dependent and SwrZ-independent mechanisms. (A) qRT-PCR measurements of transcript levels of the indicated genes in Δ*luxR* Δ*swrZ* Δ*luxT* V. harveyi harboring the indicated plasmids. pControl (black) denotes the empty parent vector, and p*luxT* (blue) and p*swrZ* (red) denote vectors harboring IPTG-inducible *luxT* and *swrZ*, respectively. RNA was isolated from strains grown for 16 h in AB medium supplemented with 0.5 mM IPTG. (B) Same as for panel A, for Δ*luxR* Δ*swrZ* V. harveyi harboring the indicated plasmids. (C) qRT-PCR measurements of transcript levels of the indicated genes in WT (black), Δ*luxT* (blue), Δ*exsA* (gray), and Δ*exsA* Δ*luxT* (white) V. harveyi strains. RNA was isolated from V. harveyi strains grown in LM medium to an OD_600_ of 0.1. (D) Model for QS and LuxT/SwrZ regulation of T3SS genes. In panels A to C, error bars represent standard deviations of the means from 3 biological replicates. In panels A and C, different letters indicate significant differences between strains (*P < *0.05 by two-way ANOVA followed by Tukey’s multiple-comparison test). In panel B, unpaired two-tailed *t* tests with Welch’s correction were performed comparing the indicated pControl and p*swrZ* conditions. *, *P* < 0.05.

To test our model, we assessed whether LuxT activates T3SS gene expression by a SwrZ-independent mechanism. To do this, we introduced a p*luxT* overexpression vector into a Δ*luxR* Δ*swrZ* Δ*luxT* V. harveyi strain. The Δ*luxR* mutation was included to eliminate HCD repression of T3SS genes. This strategy is superior to making measurements from RNA collected at LCD from strains harboring *luxR*. In the latter case, residual LuxR repressor is present (Fig. S5A). In this setup, we measured *hyp*, *vopN*, *vscO*, and *exsD* transcript levels. We also measured the expression of *exsA*, which is located immediately upstream of T3SS.4 and, as mentioned above, encodes the master transcriptional activator of T3SS genes. Compared to the empty vector control, the overexpression of *luxT* caused ∼122-, 74-, 34-, 57-, and 19-fold increases in *hyp*, *vopN*, *vscO*, *exsD*, and *exsA* expression, respectively (Fig. 4A). These data demonstrate that LuxT activates the four T3SS operons and *exsA* by a mechanism that does not require SwrZ. We also assessed *swrZ* overexpression in this experimental setup. Repression by SwrZ was not observed (Fig. 4A), presumably because in the absence of LuxT activation, T3SS gene expression is minimal, so no detectable repression by SwrZ can occur. To test this supposition, we introduced the p*swrZ* overexpression construct into a Δ*luxR* Δ*swrZ* V. harveyi strain. Indeed, in the *luxT*^+^ background, the overexpression of *swrZ* caused an ∼10-fold reduction in *hyp*, *vopN*, and *vscO* transcript levels and ∼9- and 3-fold reductions in *exsD* and *exsA* levels, respectively (Fig. 4B). Thus, SwrZ is a repressor of the four T3SS operons and *exsA*.

Previously, it was discovered that T3SS gene expression does not occur in the absence of *exsA* irrespective of the presence or absence of LuxR. Thus, ExsA is epistatic to QS in the control of T3SS genes (31). Consistent with this finding, the overexpression of *exsA* overrode repression by LuxR to activate T3SS gene expression at HCD (31). Based on these previously reported data and our identification of LuxT and SwrZ as regulators of T3SS genes, we wondered whether LuxT and SwrZ also exert control over T3SS genes via the regulation of *exsA*. To test this possibility, we measured *hyp*, *vopN*, *vscO*, and *exsD* expression in WT, Δ*luxT*, Δ*exsA*, and Δ*exsA* Δ*luxT* V. harveyi strains. Figure 4C shows that, as expected, the expression levels of all four genes were lower in the Δ*luxT* strain than in the WT. However, there were no significant differences in *hyp*, *vopN*, and *vscO* transcript levels between the Δ*exsA* and Δ*exsA* Δ*luxT* strains (Fig. 4C). We conclude that *exsA* is required for LuxT activation of T3SS.1, T3SS.2, and T3SS.3, encompassing *hyp*, *vopN*, and *vscO*, respectively. Because LuxT activation of *hyp*, *vopN*, and *vscO* requires *swrZ* (Fig. 2C), we can also conclude that regulation by SwrZ requires *exsA*. Regarding T3SS.4, there were ∼3-fold-fewer *exsD* transcripts present in the Δ*exsA* Δ*luxT* strain than in the Δ*exsA* strain. It is possible that LuxT activates the T3SS.4 operon encompassing *exsD* independently of ExsA, or alternatively, based on their sequential orientation in the genome (Fig. 2A), LuxT activates only the *exsA* promoter, and readthrough transcription occurs for T3SS.4.

Together, our results show that LuxT activates T3SS genes by two mechanisms: one is SwrZ dependent, and one is SwrZ independent. Also, LuxT functions independently of QS to control these genes. QS and LuxT/SwrZ modulate T3SS.1, T3SS.2, and T3SS.3 via control of the expression of *exsA* encoding a transcriptional activator of T3SS genes. Previous results showed that *exsA* is repressed by both AphA and LuxR, which constrains its expression to low to mid-cell densities. Our scheme for QS and LuxT/SwrZ regulation of T3SS genes is depicted in Fig. 4D.

### QS and LuxT/SwrZ regulate V. harveyi siderophore production.

**FIG 5:**
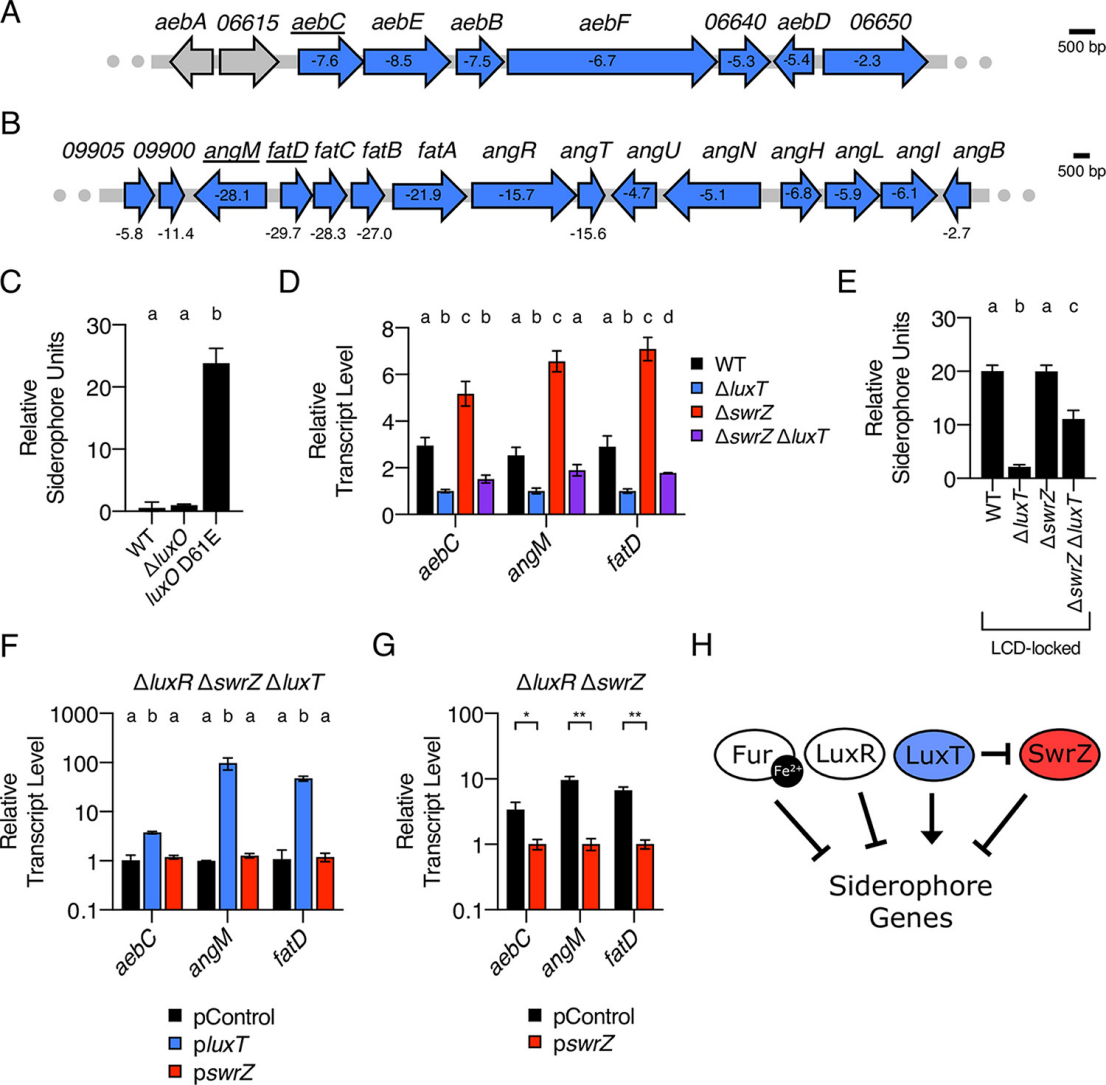
QS, LuxT, and SwrZ regulate V. harveyi siderophore production. (A and B) Schematic of siderophore gene organization in V. harveyi for amphi-enterobactin (A) and anguibactin (B). All genes labeled in blue were identified by RNA-Seq as members of the LuxT regulon. The numbers shown within or below the genes designate the fold change differences in transcript levels between WT and Δ*luxT* V. harveyi, as measured by RNA-Seq. The expression of the underlined genes was measured in panels D, F, and G. (C) CAS assay quantitation of siderophore levels in cell-free culture fluids isolated from the indicated V. harveyi strains. Strains were grown for 16 h in AB medium. (D) qRT-PCR measurements of transcript levels of the indicated genes in WT (black), Δ*luxT* (blue), Δ*swrZ* (red), and Δ*swrZ* Δ*luxT* (purple) V. harveyi strains. RNA was isolated from strains grown in LM medium to an OD_600_ of 0.1. (E) Same as for panel C. “LCD-locked” denotes that the strains harbor the *luxO* D61E mutation. (F) qRT-PCR measurements of transcript levels of the indicated genes in Δ*luxR* Δ*swrZ* Δ*luxT* V. harveyi harboring the indicated plasmids. pControl (black) denotes the empty parent vector, and p*luxT* (blue) and p*swrZ* (red) denote vectors harboring IPTG-inducible *luxT* and *swrZ*, respectively. RNA was isolated from strains grown for 16 h in AB medium supplemented with 0.5 mM IPTG. (G) Same as for panel F, for Δ*luxR* Δ*swrZ* V. harveyi harboring the indicated plasmids. (H) Model for Fur, QS, and LuxT/SwrZ regulation of V. harveyi siderophore production. In panels C to G, error bars represent standard deviations of the means from 3 biological replicates. In panels C to F, different letters indicate significant differences between strains (*P < *0.05 by two-way ANOVA followed by Tukey’s multiple-comparison test). In panel G, unpaired two-tailed *t* tests with Welch’s correction were performed comparing the indicated pControl and p*swrZ* conditions. *, *P* < 0.05; **, *P* < 0.01.

Similar to type III secretion, the RNA-Seq analysis revealed LuxT to be an activator of siderophore production genes in V. harveyi. Siderophores are small-molecule iron chelators that bacteria produce and secrete to scavenge extracellular iron (32). Companion siderophore uptake systems import siderophore-Fe^3+^ complexes, facilitating iron acquisition. V. harveyi carries the genes required to produce, secrete, and import two siderophores, amphi-enterobactin and anguibactin (33, 34), and both sets of genes are regulated by LuxT (Fig. 5A and B, respectively, and Data Set S1). V. harveyi produces siderophores at LCD, suggesting QS regulation; however, the mechanism connecting QS to siderophore genes is not defined (6, 35). Using the steps laid out above for the characterization of T3SS gene regulation, we determine how QS, LuxT, and SwrZ control siderophore production.

First, qRT-PCR validated the RNA-Seq data. The transcript levels of *aebC* encoding isochorismate synthase were measured as a representative amphi-enterobactin biosynthetic gene (33). Regarding anguibactin, the expression levels of *angM* and *fatD* were measured, and they encode a nonribosomal peptide synthetase and an anguibactin-Fe^3+^ transporter, respectively (36, 37). The transcript levels of *aebC*, *angM*, and *fatD* were ∼3-fold lower in Δ*luxT* V. harveyi than in the WT (Fig. S4C). Analogous results were obtained for the three genes when measured in Δ*qrr*1 and Δ*qrr*1 Δ*luxT* strains, showing that LuxT activates siderophore genes independently of Qrr1 (Fig. S4D).

Second, to determine the mechanism by which QS controls siderophore production, we used a chrome azurol S (CAS) dye to quantify siderophore levels in cell-free culture fluids prepared from WT V. harveyi and QS mutant strains. Approximately 24-fold more siderophore was present in fluids from the LCD-locked *luxO* D61E strain than in fluids from the WT and Δ*luxO* strains, consistent with the known LCD production pattern (Fig. 5C) (6, 35). We used mutants defective in each siderophore biosynthesis pathway to show that the siderophore detected by the CAS assay was amphi-enterobactin, not anguibactin, as follows: AebF is required for amphi-enterobactin production (33), and the *luxO* D61E Δ*aebF* strain had almost no siderophore in its cell-free culture fluids (Fig. S6A). In contrast, the *luxO* D61E Δ*angN* strain, which lacks a gene required for anguibactin production (38), did not exhibit reduced siderophore production compared to the *luxO* D61E strain (Fig. S6A). V. harveyi apparently does not produce anguibactin when grown under standard laboratory conditions, as reported previously (35).

10.1128/mbio.03621-21.6 FIG S6 LuxR and Fur repress the production of the V. harveyi amphi-enterobactin siderophore. (A to C) CAS assay quantitation of siderophore levels in cell-free culture fluids isolated from the indicated V. harveyi strains. Strains were grown for 16 h in AB medium. (D) qRT-PCR measurements of transcript levels of the indicated genes in WT (black), Δ*fur* (gray), and Δ*fur* Δ*luxT* (blue) V. harveyi strains. Strains were grown in LM medium to an OD_600_ of 0.1. (E) Same as for panel D, for WT (black), Δ*luxT* (blue), Δ*swrZ* (red), and Δ*swrZ* Δ*luxT* (purple) V. harveyi strains. In all panels, error bars represent standard deviations of the means from 3 biological replicates. In panels A, D, and E, different letters indicate significant differences between strains (*P < *0.05 by two-way ANOVA followed by Tukey’s multiple-comparison test). In panels B and C, unpaired two-tailed *t* tests with Welch’s correction were performed comparing two conditions, as indicated. ns, not significant (*P* ≥ 0.05); **, *P* < 0.01. Download FIG S6, PDF file, 0.1 MB. Copyright © 2022 Eickhoff et al. 2022 Eickhoff et al. https://creativecommons.org/licenses/by/4.0/ This content is distributed under the terms of the Creative Commons Attribution 4.0 International license.

With respect to V. harveyi QS, three mechanisms can explain how a gene acquires a LCD expression pattern (Fig. S1): it is activated at LCD by AphA; it is activated, directly or indirectly, at LCD by the Qrr sRNAs; or it is repressed at HCD by LuxR. Regarding AphA, siderophore levels were similarly high in fluids from the *luxO* D61E and *luxO* D61E Δ*aphA* V. harveyi strains, so AphA has no role in the regulation of siderophore production (Fig. S6B). Regarding LuxR, the Δ*luxR* strain possessed ∼16-fold more amphi-enterobactin in its culture fluids than did the WT at HCD (Fig. S6C). Thus, QS control of amphi-enterobactin production occurs via LuxR repression at HCD. Regarding the Qrr sRNAs, at HCD, somewhat less amphi-enterobactin was produced by the Δ*luxR* strain than by the *luxO* D61E strain (Fig. S6B and C), hinting that in addition to the primary repressive role of LuxR at HCD, at LCD, the Qrr sRNAs could activate siderophore biosynthesis or export. Possibilities include direct posttranscriptional activation by the Qrr sRNAs, or alternatively, another QS-regulated factor may exist that is involved in amphi-enterobactin gene regulation. We did not investigate this mechanism further.

To position LuxT and SwrZ in the siderophore regulatory pathway, we used qRT-PCR quantitation of *aebC*, *angM*, and *fatD* in WT, Δ*luxT*, Δ*swrZ*, and Δ*swrZ* Δ*luxT* V. harveyi strains at LCD (Fig. 5D). As shown in Fig. S4C, there were lower mRNA levels in the Δ*luxT* strain than in the WT, showing again that LuxT activates siderophore genes. Modestly increased expression of the three genes occurred in the Δ*swrZ* strain compared to the WT, indicating that SwrZ is a repressor of siderophore genes. Finally, the Δ*luxT* Δ*swrZ* double mutant expressed siderophore genes at a level intermediate between those of the Δ*luxT* and Δ*swrZ* strains. These data resemble the findings in Fig. 3A for LuxT/SwrZ regulation of T3SS genes, suggesting an identical model: LuxT activates siderophore production by both a SwrZ-dependent and a SwrZ-independent mechanism. We validate this model below.

### Fur represses V. harveyi siderophore production under iron-rich conditions.

The above-described qRT-PCR measurements of siderophore transcripts showed only modest differences between strains (Fig. 5D), possibly as a consequence of the generally low siderophore gene expression levels that occur under iron-replete conditions due to repression by Fur, the major transcriptional regulator of iron transport. Fur repression is relieved under iron-limiting conditions (39). Indeed, culture fluids used for the CAS assays in Fig. 5C and Fig. S6A to C were prepared from V. harveyi grown in low-iron minimal medium, and a significant amount of the siderophore was produced. To assess the role of Fur in the regulation of V. harveyi siderophore production, *aebC*, *angM*, and *fatD* transcript levels were compared between WT and Δ*fur* V. harveyi. In iron-rich medium, in the absence of *fur*, the expression levels of the three test genes were ∼284-, 119-, and 66-fold higher than when *fur* was present (Fig. S6D). Deletion of *luxT* in the Δ*fur* strain caused 3-, 43-, and 52-fold decreases in *aebC*, *angM*, and *fatD* transcript levels, respectively (Fig. S6D). Thus, Fur is a repressor of amphi-enterobactin and anguibactin genes, which diminished our ability to observe their regulation by LuxT/SwrZ under iron-replete conditions, particularly for *angM* and *fatD*. We note that *fur* expression is not controlled by LuxT or SwrZ (Fig. S6E). Thus, Fur and LuxT/SwrZ regulate siderophore genes independently. The siderophore experiments in the next section were conducted following the growth of V. harveyi under iron-limited conditions.

### LuxT activates V. harveyi siderophore production by SwrZ-dependent and SwrZ-independent mechanisms.

To validate the roles of and relationship between LuxT and SwrZ in the regulation of siderophore production, we quantified siderophore produced by the LCD-locked *luxO* D61E, *luxO* D61E Δ*luxT*, *luxO* D61E Δ*swrZ*, and *luxO* D61E Δ*swrZ* Δ*luxT* mutant strains using the CAS assay. Consistent with the qRT-PCR results (Fig. 5D), the *luxO* D61E Δ*swrZ* Δ*luxT* mutant produced siderophore levels intermediate between those of the *luxO* D61E Δ*luxT* strain and the *luxO* D61E Δ*swrZ* strain (Fig. 5E). These data support a model in which LuxT activates siderophore production by two mechanisms: one dependent on SwrZ and one independent of SwrZ. We performed complementation analyses in a V. harveyi Δ*luxR* Δ*luxT* Δ*swrZ* strain as a final test of our model. Even in the absence of *swrZ*, the overexpression of *luxT* caused increased expression of *aebC*, *angM*, and *fatD*, supporting the SwrZ-independent role for LuxT (Fig. 5F). Following the reasoning described above for T3SS genes, repression due to *swrZ* overexpression could be observed only in the *luxT*^+^ background (Fig. 5F and G). Our model for the regulation of siderophore production in V. harveyi is depicted in Fig. 5H: Fur represses siderophore genes under iron-replete conditions, and LuxR represses siderophore genes at HCD. LuxT activates siderophore genes by both a SwrZ-dependent and a SwrZ-independent mechanism.

### QS and LuxT/SwrZ regulate the production of the aerolysin toxin.

The final V. harveyi LuxT-regulated behavior that we investigated was the production of the pore-forming toxin aerolysin. Aerolysin-like proteins, originally discovered in the bacterium Aeromonas hydrophila, are a family of secreted proteins that form transmembrane β-barrel pores in eukaryotic target cells, causing cell death (40–42). Aerolysins are virulence factors in *Aeromonas* spp. and Vibrio splendidus (43, 44).

We previously reported that LuxT is an activator of *VIBHAR_RS11620* encoding an aerolysin toxin. For clarity, we now call *VIBHAR_RS11620 aerB*. We determined that LuxT activates *aerB* expression by two mechanisms. First, LuxT activates *aerB* transcription by a Qrr1-independent mechanism. Second, LuxT indirectly activates *aerB* translation by repressing the expression of *qrr*1 encoding a posttranscriptional repressor of *aerB* (13, 18). V. harveyi aerolysin production can be measured by monitoring the hemolysis of blood cells in liquid or on blood agar plates. Activation by LuxT is required for V. harveyi hemolytic activity in both assays. Observable repression by Qrr1 occurs only in the plate assay (18).

**FIG 6:**
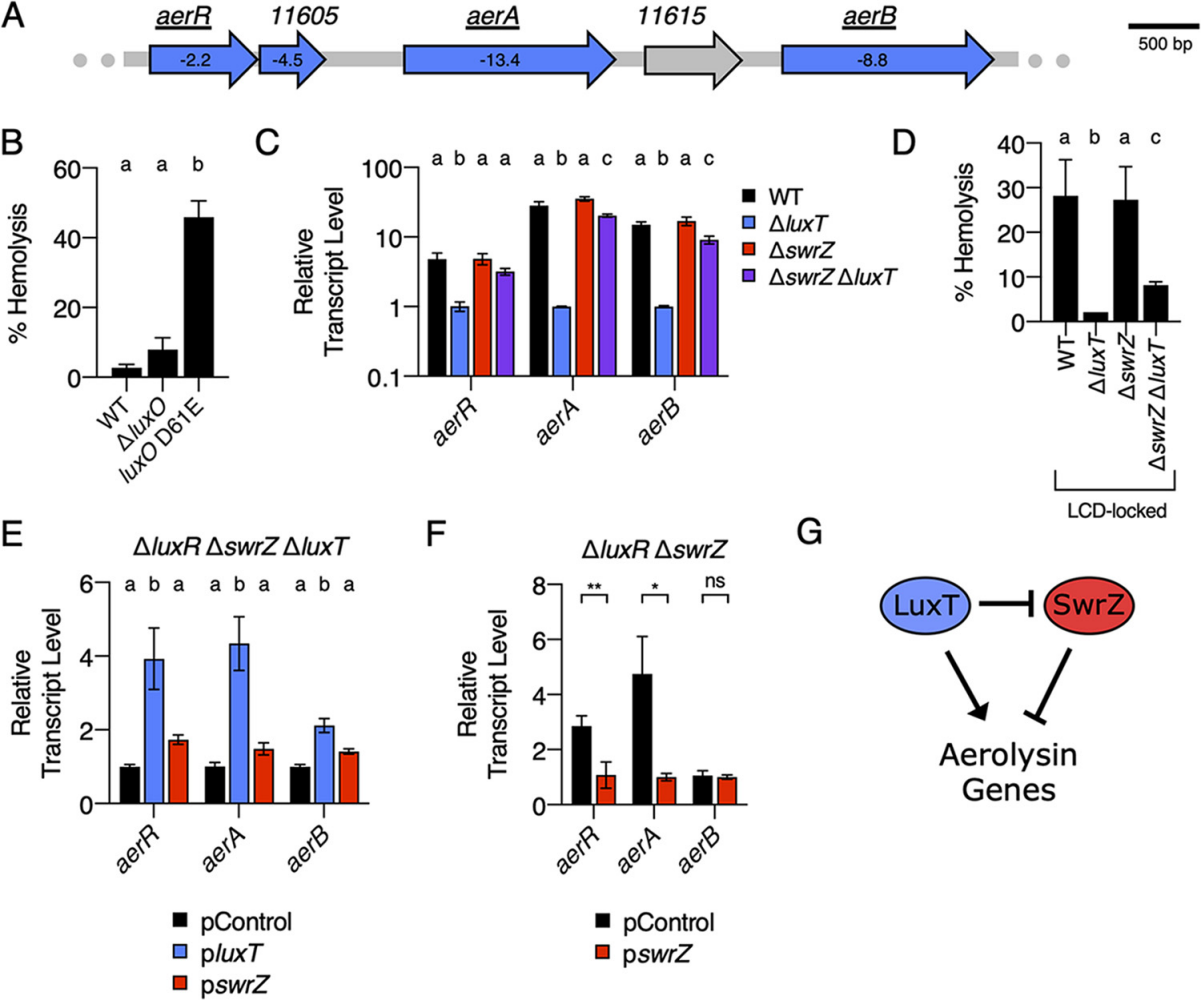
QS, LuxT, and SwrZ regulate V. harveyi aerolysin production. (A) Schematic of aerolysin gene organization in V. harveyi. All genes labeled in blue were identified by RNA-Seq as members of the LuxT regulon. The numbers shown within the genes designate the fold change differences in transcript levels between WT and Δ*luxT* V. harveyi, as measured by RNA-Seq. The expression of the underlined genes was measured in panels C, E, and F. (B) Hemolytic activity in the indicated V. harveyi cell-free culture fluids as judged by lysis of defibrinated sheep’s blood cells. Culture fluids were collected after 24 h of growth in AB medium. (C) qRT-PCR measurements of transcript levels of the indicated genes in WT (black), Δ*luxT* (blue), Δ*swrZ* (red), and Δ*swrZ* Δ*luxT* (purple) V. harveyi strains. RNA was isolated from strains grown in LM medium to an OD_600_ of 0.1. (D) Same as for panel B, for the indicated strains. (E) qRT-PCR measurements of transcript levels of the indicated genes in Δ*luxR* Δ*swrZ* Δ*luxT* V. harveyi harboring the indicated plasmids. pControl (black) denotes the empty parent vector, and p*luxT* (blue) and p*swrZ* (red) denote vectors harboring IPTG-inducible *luxT* and *swrZ*, respectively. RNA was isolated from strains grown for 16 h in AB medium supplemented with 0.5 mM IPTG. (F) Same as for panel E, for Δ*luxR* Δ*swrZ* V. harveyi harboring the indicated plasmids. (G) Model for LuxT/SwrZ regulation of aerolysin production. In panels B to F, error bars represent standard deviations of the means from 3 biological replicates. In panels B to E, different letters indicate significant differences between strains (*P < *0.05 by two-way ANOVA followed by Tukey’s multiple-comparison test). In panel F, unpaired two-tailed *t* tests with Welch’s correction were performed comparing the indicated pControl and p*swrZ* conditions. ns, not significant (*P* ≥ 0.05); *, *P* < 0.05; **, *P* < 0.01.

In agreement with our above-described findings, RNA-Seq revealed LuxT to be an activator of the *aerB* aerolysin toxin gene. Three additional genes upstream of *aerB* were also activated (Fig. 6A and Data Set S1): *VIBHAR_RS11600*, which we call *aerR*, encodes a transcriptional regulator; *VIBHAR_RS11605*, which we do not name, encodes a protein of unknown function; and *VIBHAR_RS11610*, which we call *aerA*, encodes an additional aerolysin family toxin. The protein sequences of AerA and AerB are similar (50% pairwise identity, 66% pairwise similarity), indicating a possible gene duplication event. The *VIBHAR_RS11615* gene that resides between *aerA* and *aerB* was not identified as being regulated by LuxT (Fig. 6A).

As described above, we first confirmed LuxT regulation of *aerR*, *aerA*, and *aerB* and determined if Qrr1 is required. Deletion of *luxT* in WT V. harveyi resulted in ∼5-, 28-, and 15-fold-lower expression levels of *aerR*, *aerA*, and *aerB*, respectively (Fig. S4E). In a strain lacking *qrr*1, deletion of *luxT* caused similar reductions in expression (Fig. S4F). These data show that Qrr1 is not required for LuxT activation of aerolysin genes. We know from our previous work that Qrr1 is a posttranscriptional regulator of *aerB*. Regulation by Qrr1 can be detected only when measuring *aerB* translation, not transcription (13, 18). For the remainder of the present work, we focus on the transcriptional, Qrr1-independent mechanism by which LuxT activates aerolysin production.

QS regulation of aerolysin production is more complicated than we anticipated; nonetheless, we lay out what we know here. In the liquid hemolysis assay, culture fluids from the LCD-locked *luxO* D61E strain possessed ∼17-fold more hemolysis activity than fluids from HCD WT V. harveyi (Fig. 6B). Fluids from the HCD-locked Δ*luxO* strain had activity similar to that of the WT strain (Fig. 6B). Thus, aerolysin production is QS controlled and produced at LCD. Deletion of *aphA* in the *luxO* D61E LCD-locked strain did not reduce hemolysis (Fig. S7A), and deletion of *luxR* in the WT did not increase hemolysis at HCD (Fig. S7B). These data were unexpected and show that while aerolysin is QS controlled, neither AphA nor LuxR regulates its production. We confirmed the results using blood agar plates. The results on the plates were identical to those in liquid with the exception that the Δ*luxR* strain caused less hemolysis on plates than did the WT (Fig. S7C). Given the LCD production pattern for aerolysin, we conclude that aerolysin genes must be activated at LCD by the QS Qrr sRNAs, either directly or indirectly. We note that this finding seemingly contradicts our previous result showing that Qrr sRNAs posttranscriptionally repress *aerB* (13, 18). We preliminarily predict that the Qrr sRNAs exert both positive and negative effects on aerolysin production. Activation by the Qrr sRNAs is likely indirect through an unknown regulator, while posttranscriptional repression of *aerB* occurs directly (13). Further investigation to characterize the mechanism of QS control of aerolysin genes is required.

10.1128/mbio.03621-21.7 FIG S7 Assessment of the requirements for V. harveyi aerolysin production. (A, B, and D) Hemolytic activity present in the indicated V. harveyi cell-free culture fluids as judged by lysis of defibrinated sheep’s blood cells. Culture fluids were collected after 24 h of growth in AB medium. (C, E, and F) Hemolytic activity produced by the indicated V. harveyi strains as judged by halo formation on tryptic soy agar (TSA) plates containing 5% sheep’s blood. Strains were grown for 72 h at 30°C. In panels A, B, and D, error bars represent standard deviations of the means from 3 biological replicates. In panels A and B, unpaired two-tailed *t* tests with Welch’s correction were performed comparing two conditions, as indicated. ns, not significant (*P* ≥ 0.05). In panel D, different letters indicate significant differences between strains (*P < *0.05 by two-way ANOVA followed by Tukey’s multiple-comparison test). Download FIG S7, TIF file, 1.4 MB. Copyright © 2022 Eickhoff et al. 2022 Eickhoff et al. https://creativecommons.org/licenses/by/4.0/ This content is distributed under the terms of the Creative Commons Attribution 4.0 International license.

While the mechanism underlying QS regulation of aerolysin genes is not fully defined, what is clear is that *aerR*, *aerA*, and *aerB* are all regulated by LuxT (Data Set S1 and Fig. S4E). Thus, we next explored which of the three genes is required for V. harveyi hemolysis activity. We deleted each gene individually in the LCD-locked *luxO* D61E V. harveyi strain. Deletion of *aerR* and *aerA* eliminated hemolysis activity in the liquid and plate assays (Fig. S7D and E, respectively). In contrast, deletion of *aerB* did not reduce hemolytic activity, so *aerB* is not required (Fig. S7D and E). We speculate that AerR is an activator of *aerA*, and AerA is the primary secreted aerolysin toxin. Because *aerB* is repressed by the Qrr sRNAs, the *aerB* expression level may remain low at LCD.

To understand the mechanism underlying LuxT activation of aerolysin gene expression, we measured *aerR*, *aerA*, and *aerB* transcript levels in WT, Δ*luxT*, Δ*swrZ*, and Δ*swrZ* Δ*luxT* V. harveyi strains at LCD. The expression levels of all three genes were high in the WT and Δ*swrZ* strains, low in the Δ*luxT* strain, and intermediate in the Δ*swrZ* Δ*luxT* strain (Fig. 6C). The corresponding hemolysis patterns in the LCD-locked *luxO* D61E background exactly mirrored the transcription patterns both in the liquid assay (Fig. 6D) and on the blood agar plates (Fig. S7F). Exactly analogous to what we have explained above, these data indicate that LuxT activates aerolysin production by both a SwrZ-dependent and a SwrZ-independent mechanism. We verified this assumption using complementation analyses (Fig. 6E and F). The results were as expected according to our putative mechanism except that *swrZ* overexpression did not repress *aerB* (Fig. 6F). However, the qRT-PCR results in Fig. 6C show that SwrZ is a repressor of *aerB*. We presume that *swrZ* complementation did not repress *aerB* because *aerB* exhibits quite low basal expression levels. Our model for LuxT/SwrZ regulation of aerolysin production is depicted in Fig. 6G.

### *luxT* is and *swrZ* is not conserved among *Vibrionaceae* family members.

As mentioned above, SwrT (LuxT) repression of *swrZ* was originally discovered in V. parahaemolyticus and shown to be relevant for the regulation of swarming motility (22). Here, we have shown that LuxT also regulates *swrZ* in V. harveyi, and both LuxT and SwrZ regulate type III secretion, siderophore production, and aerolysin production. LuxT also regulates genes in V. harveyi that SwrZ does not control. For example, LuxT activates *luxCDABE*, encoding luciferase, and SwrZ plays no role (18, 20) (Fig. S8). We assessed SwrZ involvement in the regulation of 5 additional LuxT-activated and 3 additional LuxT-repressed genes. SwrZ regulated all 5 of the LuxT-activated test genes, whereas SwrZ did not regulate the 3 LuxT-repressed test genes (Fig. S8). Based on the opposing roles of LuxT and SwrZ (Fig. 3B to E), we speculate that SwrZ may function only as a transcriptional repressor, possibly explaining why it is not required to participate in LuxT repression of gene expression. We conclude that within the LuxT regulon, a subset of genes does not employ SwrZ in regulation.

10.1128/mbio.03621-21.8 FIG S8 LuxT regulates genes by SwrZ-dependent and SwrZ-independent mechanisms in V. harveyi. Shown are qRT-PCR measurements of transcript levels of the indicated genes in WT (black), Δ*luxT* (blue), Δ*swrZ* (red), and Δ*swrZ* Δ*luxT* (purple) V. harveyi strains. Strains were grown in LM medium to an OD_600_ of 0.1. Error bars represent standard deviations of the means from 3 biological replicates. Different letters indicate significant differences between strains (*P < *0.05 by two-way ANOVA followed by Tukey’s multiple-comparison test). Download FIG S8, PDF file, 0.03 MB. Copyright © 2022 Eickhoff et al. 2022 Eickhoff et al. https://creativecommons.org/licenses/by/4.0/ This content is distributed under the terms of the Creative Commons Attribution 4.0 International license.

**FIG 7:**
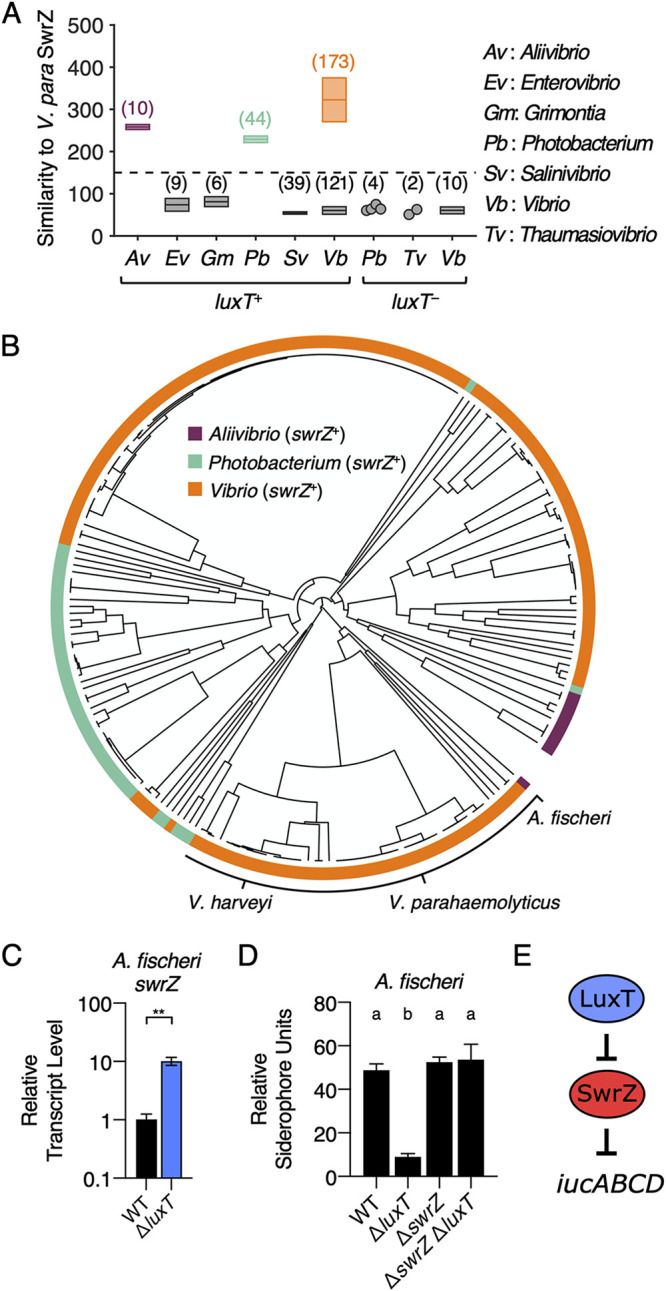
Cooccurrence of *luxT* and *swrZ* genes among *Vibrionaceae* species. (A) Highest similarity in protein sequences (BLOSUM62) in the indicated genera to V. parahaemolyticus SwrZ. Species are divided into two groups, one possessing *luxT* (*luxT*^+^) and one lacking *luxT* (*luxT*^−^). The dashed line indicates the cutoff used for the similarity score to differentiate *swrZ*^+^ from *swrZ*^−^ strains. Gray indicates groups of species that lack *swrZ*. The numbers in parentheses indicate the number of species in each group. Boxes show the means ± standard deviations for groups with >5 species. Circles represent individual species in groups with ≤5 members. Purple, green, and orange denote *Aliivibrio*, *Photobacterium*, and *Vibrio* species that possess *swrZ*, respectively. (B) Phylogenetic tree based on the *swrZ* promoter regions in *Vibrionaceae*. Phylogenetic distances were computed based on sequences spanning positions −122 to −8, relative to the *swrZ* start codons. Colors are the same as those in panel A. (C) qRT-PCR measurements of *swrZ* transcript levels in WT (black) and Δ*luxT* (blue) A. fischeri. RNA was isolated from strains grown in LM medium to an OD_600_ of 0.1. (D) CAS assay quantitation of siderophore levels in cell-free culture fluids prepared from the indicated A. fischeri strains. Strains were grown for 16 h in AB medium. (E) Model for LuxT/SwrZ regulation of A. fischeri aerobactin siderophore production. In panels C and D, error bars represent standard deviations of the means from 3 biological replicates. In panel C, an unpaired two-tailed *t* test with Welch’s correction was performed comparing the indicated WT and Δ*luxT* conditions. **, *P* < 0.01. In panel D, different letters indicate significant differences between strains (*P < *0.05 by two-way ANOVA followed by Tukey’s multiple-comparison test).

In V. harveyi and V. parahaemolyticus, the *luxT* (*swrT*) and *swrZ* genes are carried on the two different chromosomes, so while they coregulate many genes, it is unlikely that they were inherited as a pair. Knowing their interconnected roles in V. harveyi and V. parahaemolyticus, we wondered whether LuxT and SwrZ jointly regulate functions in other *Vibrionaceae* species. To garner preliminary evidence for or against this possibility, we examined the conservation of both genes within the *Vibrionaceae* family. Among the 418 sequenced *Vibrionaceae* species, the *luxT* gene is present in all but 16 species (96%) (Fig. 7A). None of the 16 species lacking *luxT* carries a *swrZ* gene (Fig. 7A). Among the 402 *Vibrionaceae* species that possess *luxT*, 227 species (56%) also have *swrZ* (Fig. 7A); these genera include *Aliivibrio*, *Photobacterium*, and *Vibrio* (Fig. 7A). Thus, in cases in which both *luxT* and *swrZ* are present, the possibility of coregulation of target genes exists. In the species that have *luxT* but lack *swrZ*, LuxT must regulate genes independently of SwrZ. Together, these observations may explain the evolution of SwrZ-dependent and SwrZ-independent functions for LuxT in V. harveyi.

To probe the possibility that LuxT also represses *swrZ* in other species, we performed a phylogenetic analysis by comparing the *swrZ* promoter regions of all *luxT^+^ swrZ*^+^ species. Our prediction was that *swrZ* promoters would be more similar in species in which LuxT represses *swrZ* than in species in which LuxT does not regulate *swrZ*. In support of this idea, the V. harveyi and V. parahaemolyticus *swrZ* promoter sequences are indeed similar. Thus, when we constructed a phylogenetic tree based on *swrZ* promoter sequences, V. harveyi and V. parahaemolyticus reside in proximity (Fig. 7B). As an initial test of LuxT regulation of *swrZ* in a species beyond V. harveyi and V. parahaemolyticus, we analyzed LuxT repression of *swrZ* in Aliivibrio fischeri, a species that neighbors V. harveyi and V. parahaemolyticus on the *swrZ* promoter phylogenetic tree (Fig. 7B). Indeed, *swrZ* transcript levels were ∼10-fold higher in Δ*luxT* A. fischeri than in the WT, showing that LuxT is a repressor of *swrZ* in A. fischeri (Fig. 7C). We previously reported that LuxT is an indirect activator of *iucABCD*, encoding the aerobactin siderophore biosynthesis genes in A. fischeri (21). At that time, we did not know the identity of the regulator connecting LuxT to *iucABCD*. Our findings here point to SwrZ fulfilling that function. To test this idea, we used the CAS assay to measure siderophore levels in culture fluids from WT, Δ*luxT*, Δ*swrZ*, and Δ*swrZ* Δ*luxT* A. fischeri strains. Indeed, LuxT activates A. fischeri siderophore production only in the *swrZ*^+^ background, as the deletion of *luxT* in the Δ*swrZ* strain caused no change in siderophore production (Fig. 7D). Thus, LuxT activates A. fischeri siderophore production via a SwrZ-dependent mechanism. There is no evidence that a second SwrZ-independent mechanism occurs (Fig. 7D). Our model for LuxT and SwrZ regulation of A. fischeri siderophore production is depicted in Fig. 7E.

## DISCUSSION

QS controls over 600 genes in V. harveyi (8). Integral to the V. harveyi QS process are five, largely redundant, Qrr sRNAs that posttranscriptionally regulate target gene expression. LuxT is a TetR family transcriptional regulator that was previously identified to repress the expression of V. harveyi *qrr*1 encoding the Qrr1 sRNA (18). When LuxT acts as a *qrr*1 repressor, it tunes the expression of select Qrr1-controlled genes that are members of the much larger QS regulon. Here, we use RNA-Seq to discover that beyond controlling *qrr*1 expression, LuxT is a global regulator in V. harveyi that controls 414 genes. Analogous to what has been reported for V. parahaemolyticus (22), LuxT directly represses *swrZ*, encoding an additional transcriptional regulator. We show that in V. harveyi, LuxT activates type III secretion, siderophore production, and aerolysin production genes independently of Qrr1 by both SwrZ-dependent and SwrZ-independent mechanisms. In the future, defining the SwrZ regulon will be a crucial step in gaining a comprehensive understanding of the individual and combined roles of LuxT and SwrZ in regulating V. harveyi gene expression.

Two feedback loops exist in the V. harveyi LuxT/SwrZ regulatory pathway: LuxT and SwrZ activate and repress, respectively, their own transcription. Generally, positive feedback is thought to amplify responses to a stimulus, whereas negative feedback buffers pathway output against fluctuations in stimuli (45, 46). Thus, the inclusion of both positive and negative feedback in a single system can alter both input sensitivity and output dynamics (46, 47). We speculate that the LuxT positive feedback loop enables V. harveyi to rapidly commit to the LuxT “on” state following the initial activation of *luxT* expression. In contrast, SwrZ-directed negative feedback on *swrZ*, which occurs when LuxT is in the “off” state, should prevent runaway *swrZ* expression. Moreover, LuxT and SwrZ have opposing regulatory functions, so genes activated by LuxT are repressed by SwrZ. Negative SwrZ autoregulation could, by tamping down SwrZ production, accelerate the expression of LuxT-activated genes when V. harveyi transitions to the LuxT “on” state.

Our analyses revealed that LuxT activates T3SS, siderophore, and aerolysin genes by two mechanisms, one SwrZ dependent and one SwrZ independent. We propose two advantages to this regulatory arrangement. First, this dual mechanism may promote a “switch-like” response. Because SwrZ represses genes that are activated by LuxT, when LuxT is in the “on” state, stronger target gene activation will occur if *swrZ* is repressed by LuxT than if not. Second, if the expression and/or activity of either or both LuxT and SwrZ responds to additional regulatory inputs, linking the two nodes could promote more nuanced control of gene expression than if LuxT and SwrZ were not connected in the circuit.

For V. harveyi to transition between LuxT “on” and LuxT “off” states, a cue(s) must activate *luxT* expression or increase LuxT activity. We do not know the identity of this putative cue. We do know that LuxT is not regulated by QS, and consistent with this finding, *luxT* expression remains steady over the V. harveyi growth curve (see Fig. S3A and B in the supplemental material). Curiously, however, genes in the LuxT-controlled regulon overlap those in the QS-controlled regulon. In particular, LuxT activates the expression of type III secretion, siderophore production, and aerolysin production genes, all of which are also QS controlled and expressed at LCD, which is the typical pattern for QS-controlled virulence genes in vibrios (48). The above-mentioned QS- and LuxT-regulated traits are all virulence factors that contribute to the success of vibrios as pathogens in host organisms (43, 49, 50). For example, in shrimp larvae, V. harveyi T3SS gene expression is 1,000-fold higher than that in laboratory culture (49). Possibly, *luxT* expression or LuxT activity is modulated by a host factor. Alternatively, or in addition, TetR family members frequently bind small-molecule ligands (51). It could be that a V. harveyi*-*, host-, or environment-derived ligand promotes heightened LuxT activity. Future studies investigating the regulation of *luxT* expression or LuxT activity should define the connection between the LCD state and LuxT function.

Our phylogenetic analyses revealed that *luxT*, but not *swrZ*, is conserved among *Vibrionaceae*, and LuxT repression of *swrZ* is conserved at least in V. parahaemolyticus, V. harveyi, and our A. fischeri test case (22). Going forward, it will be interesting to learn how LuxT function has diverged in species that harbor *luxT* but lack *swrZ*, such as in Vibrio cholerae C6706. RNA-Seq experiments similar to those described here could be used to begin to parse the roles that LuxT plays and, based on the findings, paint a coherent evolutionary picture of LuxT control of *Vibrionaceae* biology.

## MATERIALS AND METHODS

### Bacterial strains, culture conditions, and standard methods.

All strains are listed in Table S1A in the supplemental material. V. harveyi strains were derived from V. harveyi BB120 (ATCC BAA-1116) (52). Previously, V. harveyi BB120 was reclassified as Vibrio campbellii BB120 (53). For literary consistency, we refer to this strain as V. harveyi. A. fischeri strains were derived from A. fischeri ES114 (54). E. coli S17-1 λ*pir* was used for cloning. E. coli MG1655 was used for heterologous gene expression. V. harveyi and A. fischeri strains were grown in either Luria marine (LM) medium or minimal autoinducer bioassay (AB) medium at 30°C with shaking (55, 56). AB medium contained 0.4% vitamin-free Casamino Acids (Difco). E. coli strains were grown in LB medium at 37°C for cloning or at 30°C for heterologous gene expression. When necessary, kanamycin, chloramphenicol, ampicillin, and polymyxin B were added at 100 μg mL^−1^, 10 μg mL^−1^, 100 μg mL^−1^, and 50 μg mL^−1^, respectively. Gene expression from the P*_BAD_* and P*_tac_* promoters was induced following the addition of 0.01% arabinose and 0.5 mM isopropyl β-d-1-thiogalactopyranoside (IPTG), respectively, at the time of inoculation. Procedures for LuxT-6×His purification, EMSAs, qRT-PCR measurements, and hemolysis assays were described previously (18), as were CAS siderophore detection assays (21). Primers used to amplify DNA probes for EMSAs and for qRT-PCR measurements are listed in Table S1B. Growth conditions for qRT-PCR experiments are provided in the figure legends.

10.1128/mbio.03621-21.9 TABLE S1 Strains, primers, and plasmids used in this study. (A) Strain list; (B) primer list; (C) plasmid list. Download Table S1, DOCX file, 0.05 MB. Copyright © 2022 Eickhoff et al. 2022 Eickhoff et al. https://creativecommons.org/licenses/by/4.0/ This content is distributed under the terms of the Creative Commons Attribution 4.0 International license.

### DNA manipulation and strain construction.

Oligonucleotide primers were purchased from Integrated DNA Technologies (IDT) and are listed in Table S1B. PCR reactions contained either KOD Hot Start DNA polymerase (Sigma) or iProof DNA polymerase (Bio-Rad). All cloning was completed using Gibson assembly master mix (New England BioLabs) for isothermal DNA assembly (57). Plasmids were verified by sequencing (Genewiz) and are listed in Table S1C. Regarding the nomenclature of our constructs, a capital P designates the promoter driving transcription (e.g., P*_swrZ_*-*lux*). Plasmids that promote the overexpression of genes are designated with a lowercase p (e.g., p*swrZ*). The P*_swrZ_*-*lux* and P*_luxT_*-*lux* transcriptional reporters included 114- and 531-bp promoter regions, respectively, to drive the transcription of *luxCDABEG*. A consensus ribosome-binding site was included in both reporters to drive translation. Plasmids were introduced into E. coli by electroporation using a Bio-Rad MicroPulser. Plasmids were conjugated into V. harveyi and A. fischeri from E. coli S17-1 λ*pir*. V. harveyi exconjugants were selected on agar plates containing polymyxin B, and A. fischeri exconjugants were selected on agar plates containing ampicillin. Chromosomal alterations in V. harveyi and A. fischeri were introduced using the pRE112 vector harboring the *sacB* counterselectable marker as previously described (18, 58, 59). Mutations were verified by PCR and/or sequencing.

### RNA sequencing.

Cells from cultures of WT and Δ*luxT* V. harveyi strains grown overnight in LM medium were pelleted by centrifugation at 21,100 × *g* (Eppendorf 5424 centrifuge) and resuspended in fresh LM medium. Fresh cultures containing 25 mL of LM medium were inoculated with the washed cells at an OD_600_ of 0.005. The cultures were incubated at 30°C with shaking, and RNA was isolated from 3 biological replicates of each strain when the cultures had reached an OD_600_ of 0.1 using the RNeasy minikit (catalog number 74106; Qiagen). RNA-Seq was performed at the Genomics Core Facility at Princeton University, as previously described (60). Reads were mapped to the V. harveyi BB120 (ATCC BAA-1116) genome using TopHat (61). Genes with differential expression were identified using DESeq2 (62), and those exhibiting a log_2_ fold change in expression >1, along with a *P* value <0.01, in the Δ*luxT* strain compared to WT V. harveyi were designated members of the LuxT regulon.

### Bioluminescence assays.

E. coli harboring the P*_swrZ_*-*lux* reporter or V. harveyi harboring the P*_luxT_*-*lux* reporter were grown overnight, pelleted by centrifugation at 21,100 × *g* (Eppendorf 5424 centrifuge), and resuspended in LB or LM medium, respectively. The E. coli and V. harveyi cells were inoculated into fresh LB and LM medium, respectively, with normalization to a starting OD_600_ of 0.005. A total of 150 μL of each culture was transferred to wells of a 96-well plate (Corning) in quadruplicate technical replicates and overlaid with 50 μL of mineral oil (Sigma). The plates were incubated with shaking at 30°C, and the bioluminescence and OD_600_ were measured every 15 min for 24 h using a BioTek Synergy Neo2 multimode reader (BioTek, Winooski, VT, USA). Relative light units (RLU) (bioluminescence/OD_600_) represent the values when each sample was at an OD_600_ of 1.

### VopD Western blot analyses.

Cytoplasmic VopD levels were measured in V. harveyi that had been grown in AB medium supplemented with 5 mM EGTA as previously described (5). Cells equivalent to 1 OD_600_ unit were pelleted by centrifugation at 21,100 × *g* (Eppendorf 5424 centrifuge), resuspended in 100 μL of SDS-PAGE sample buffer (Bio-Rad), and boiled for 10 min. Samples were loaded onto 4–20% Mini-Protean TGX gels (Bio-Rad) and subjected to electrophoresis for 30 min at 50 mA. Following Western transfer, nitrocellulose membranes were cut in half. One portion was probed with an antibody against VopD, and the other portion was probed with an antibody against the LuxS control, as previously described (5). An anti-rabbit IgG(H+L) horseradish peroxidase (HRP) conjugate (Promega) was used as the secondary antibody. Proteins were visualized using SuperSignal West Femto maximum-sensitivity substrate (Thermo Fisher) and an ImageQuant LAS 4000 imager.

### Phylogenetic analyses.

Genomic DNA sequences of 418 *Vibrionaceae* species were downloaded from the GenBank database (63). Genes encoding *luxT* among *Vibrionaceae* species were identified previously (18). A custom MATLAB (2020; MathWorks) search algorithm based on protein sequence similarity was used to identify genes encoding SwrZ. Briefly, protein sequences of SwrZ from V. harveyi ATCC BAA-1116 and V. parahaemolyticus RIMD 2210633 were used as queries. Both yielded similar search results. The chromosomes or contigs of species under consideration were first converted to amino acid sequences and subsequently scanned for regions similar to the query sequences. To identify regions of highest similarity between protein sequences, local sequence alignments were performed using the Smith-Waterman (SW) algorithm (64). The standard substitution matrix BLOSUM62 (https://ftp.ncbi.nih.gov/blast/matrices/) was used to compute similarity score, *S*, which considers both the length and sequence similarity of the alignment. A cutoff *S* value of >150 was used to identify putative genes encoding SwrZ. Genes identified as *swrZ* homologs were verified to encode GntR family transcriptional regulators. Species lacking *swrZ* were excluded from further phylogenetic analyses. The protocols used to perform phylogenetic analyses and tree building were described previously (18).

### Data availability.

All relevant data are within the manuscript and the supplemental material. RNA-Seq data and files containing the numerical data for the main text and supplemental figures are provided at Zenodo (https://doi.org/10.5281/zenodo.5719716).

## References

[1] Bervoets I, Charlier D. 2019. Diversity, versatility and complexity of bacterial gene regulation mechanisms: opportunities and drawbacks for applications in synthetic biology. FEMS Microbiol Rev 43:304–339. doi:10.1093/femsre/fuz001. 30721976 PMC6524683

[2] Gottesman S. 2019. Trouble is coming: signaling pathways that regulate general stress responses in bacteria. J Biol Chem 294:11685–11700. doi:10.1074/jbc.REV119.005593. 31197038 PMC6682744

[3] Papenfort K, Bassler BL. 2016. Quorum sensing signal-response systems in Gram-negative bacteria. Nat Rev Microbiol 14:576–588. doi:10.1038/nrmicro.2016.89. 27510864 PMC5056591

[4] Waters CM, Bassler BL. 2005. Quorum sensing: cell-to-cell communication in bacteria. Annu Rev Cell Dev Biol 21:319–346. doi:10.1146/annurev.cellbio.21.012704.131001. 16212498

[5] Henke JM, Bassler BL. 2004. Quorum sensing regulates type III secretion in *Vibrio harveyi* and *Vibrio parahaemolyticus*. J Bacteriol 186:3794–3805. doi:10.1128/JB.186.12.3794-3805.2004. 15175293 PMC419960

[6] Lilley BN, Bassler BL. 2000. Regulation of quorum sensing in *Vibrio harveyi* by LuxO and sigma-54. Mol Microbiol 36:940–954. doi:10.1046/j.1365-2958.2000.01913.x. 10844680

[7] Henke JM, Bassler BL. 2004. Three parallel quorum-sensing systems regulate gene expression in *Vibrio harveyi*. J Bacteriol 186:6902–6914. doi:10.1128/JB.186.20.6902-6914.2004. 15466044 PMC522208

[8] van Kessel JC, Rutherford ST, Shao Y, Utria AF, Bassler BL. 2013. Individual and combined roles of the master regulators AphA and LuxR in control of the *Vibrio harveyi* quorum-sensing regulon. J Bacteriol 195:436–443. doi:10.1128/JB.01998-12. 23204455 PMC3554009

[9] Lenz DH, Mok KC, Lilley BN, Kulkarni RV, Wingreen NS, Bassler BL. 2004. The small RNA chaperone Hfq and multiple small RNAs control quorum sensing in *Vibrio harveyi* and *Vibrio cholerae*. Cell 118:69–82. doi:10.1016/j.cell.2004.06.009. 15242645

[10] Tu KC, Bassler BL. 2007. Multiple small RNAs act additively to integrate sensory information and control quorum sensing in *Vibrio harveyi*. Genes Dev 21:221–233. doi:10.1101/gad.1502407. 17234887 PMC1770904

[11] Shao Y, Bassler BL. 2012. Quorum-sensing non-coding small RNAs use unique pairing regions to differentially control mRNA targets. Mol Microbiol 83:599–611. doi:10.1111/j.1365-2958.2011.07959.x. 22229925 PMC3262071

[12] Rutherford ST, van Kessel JC, Shao Y, Bassler BL. 2011. AphA and LuxR/HapR reciprocally control quorum sensing in vibrios. Genes Dev 25:397–408. doi:10.1101/gad.2015011. 21325136 PMC3042162

[13] Shao Y, Feng L, Rutherford ST, Papenfort K, Bassler BL. 2013. Functional determinants of the quorum-sensing non-coding RNAs and their roles in target regulation. EMBO J 32:2158–2171. doi:10.1038/emboj.2013.155. 23838640 PMC3730234

[14] Tu KC, Long T, Svenningsen SL, Wingreen NS, Bassler BL. 2010. Negative feedback loops involving small regulatory RNAs precisely control the *Vibrio harveyi* quorum-sensing response. Mol Cell 37:567–579. doi:10.1016/j.molcel.2010.01.022. 20188674 PMC2844700

[15] Teng S-W, Schaffer JN, Tu KC, Mehta P, Lu W, Ong NP, Bassler BL, Wingreen NS. 2011. Active regulation of receptor ratios controls integration of quorum-sensing signals in *Vibrio harveyi*. Mol Syst Biol 7:491. doi:10.1038/msb.2011.30. 21613980 PMC3130561

[16] Freeman JA, Lilley BN, Bassler BL. 2000. A genetic analysis of the functions of LuxN: a two-component hybrid sensor kinase that regulates quorum sensing in *Vibrio harveyi*. Mol Microbiol 35:139–149. doi:10.1046/j.1365-2958.2000.01684.x. 10632884

[17] Freeman JA, Bassler BL. 1999. A genetic analysis of the function of LuxO, a two-component response regulator involved in quorum sensing in *Vibrio harveyi*. Mol Microbiol 31:665–677. doi:10.1046/j.1365-2958.1999.01208.x. 10027982

[18] Eickhoff MJ, Fei C, Huang X, Bassler BL. 2021. LuxT controls specific quorum-sensing-regulated behaviors in *Vibrionaceae* spp. via repression of *qrr*1, encoding a small regulatory RNA. PLoS Genet 17:e1009336. doi:10.1371/journal.pgen.1009336. 33793568 PMC8043402

[19] Lin YH, Miyamoto C, Meighen EA. 2000. Purification and characterization of a *luxO* promoter binding protein LuxT from *Vibrio harveyi*. Protein Expr Purif 20:87–94. doi:10.1006/prep.2000.1285. 11035955

[20] Lin YH, Miyamoto C, Meighen EA. 2000. Cloning and functional studies of a *luxO* regulator LuxT from *Vibrio harveyi*. Biochim Biophys Acta 1494:226–235. doi:10.1016/s0167-4781(00)00236-0. 11121579

[21] Eickhoff MJ, Bassler BL. 2020. *Vibrio fischeri* siderophore production drives competitive exclusion during dual-species growth. Mol Microbiol 114:244–261. doi:10.1111/mmi.14509. 32259318 PMC7541421

[22] Jaques S, McCarter LL. 2006. Three new regulators of swarming in *Vibrio parahaemolyticus*. J Bacteriol 188:2625–2635. doi:10.1128/JB.188.7.2625-2635.2006. 16547050 PMC1428401

[23] Liu H, Gu D, Cao X, Liu Q, Wang Q, Zhang Y. 2012. Characterization of a new quorum sensing regulator *luxT* and its roles in the extracellular protease production, motility, and virulence in fish pathogen *Vibrio alginolyticus*. Arch Microbiol 194:439–452. doi:10.1007/s00203-011-0774-x. 22130678

[24] Petersen BD, Liu MS, Podicheti R, Yang AY-P, Simpson CA, Hemmerich C, Rusch DB, van Kessel JC. 2021. The polar flagellar transcriptional regulatory network in *Vibrio campbellii* deviates from canonical *Vibrio* species. J Bacteriol 203:e00276-21. doi:10.1128/JB.00276-21. PMC8459767 34339299

[25] Green ER, Mecsas J. 2016. Bacterial secretion systems: an overview. Microbiol Spectr 4:VMBF-0012-2015. doi:10.1128/microbiolspec.VMBF-0012-2015. PMC4804464 26999395

[26] Deng W, Marshall NC, Rowland JL, McCoy JM, Worrall LJ, Santos AS, Strynadka NCJ, Finlay BB. 2017. Assembly, structure, function and regulation of type III secretion systems. Nat Rev Microbiol 15:323–337. doi:10.1038/nrmicro.2017.20. 28392566

[27] Galán JE, Lara-Tejero M, Marlovits TC, Wagner S. 2014. Bacterial type III secretion systems: specialized nanomachines for protein delivery into target cells. Annu Rev Microbiol 68:415–438. doi:10.1146/annurev-micro-092412-155725. 25002086 PMC4388319

[28] Morot A, El Fekih S, Bidault A, Le Ferrand A, Jouault A, Kavousi J, Bazire A, Pichereau V, Dufour A, Paillard C, Delavat F. 2021. Virulence of *Vibrio harveyi *ORM4 towards the European abalone *Haliotis tuberculata* involves both quorum sensing and a type III secretion system. Environ Microbiol 23:5273–5288. doi:10.1111/1462-2920.15592. 33989448

[29] Park K-S, Ono T, Rokuda M, Jang M-H, Okada K, Iida T, Honda T. 2004. Functional characterization of two type III secretion systems of *Vibrio parahaemolyticus*. Infect Immun 72:6659–6665. doi:10.1128/IAI.72.11.6659-6665.2004. 15501799 PMC523034

[30] Zeb S, Shah MA, Yasir M, Awan HM, Prommeenate P, Klanchui A, Wren BW, Thomson N, Bokhari H. 2019. Type III secretion system confers enhanced virulence in clinical non-O1/non-O139 *Vibrio cholerae*. Microb Pathog 135:103645. doi:10.1016/j.micpath.2019.103645. 31356927

[31] Waters CM, Wu JT, Ramsey ME, Harris RC, Bassler BL. 2010. Control of the type 3 secretion system in *Vibrio harveyi* by quorum sensing through repression of ExsA. Appl Environ Microbiol 76:4996–5004. doi:10.1128/AEM.00886-10. 20543047 PMC2916497

[32] Neilands JB. 1995. Siderophores: structure and function of microbial iron transport compounds. J Biol Chem 270:26723–26726. doi:10.1074/jbc.270.45.26723. 7592901

[33] Zane HK, Naka H, Rosconi F, Sandy M, Haygood MG, Butler A. 2014. Biosynthesis of amphi-enterobactin siderophores by *Vibrio harveyi* BAA-1116: identification of a bifunctional nonribosomal peptide synthetase condensation domain. J Am Chem Soc 136:5615–5618. doi:10.1021/ja5019942. 24701966

[34] Naka H, Actis LA, Crosa JH. 2013. The anguibactin biosynthesis and transport genes are encoded in the chromosome of *Vibrio harveyi*: a possible evolutionary origin for the pJM1 plasmid-encoded system of *Vibrio anguillarum*? Microbiologyopen 2:182–194. doi:10.1002/mbo3.65. 23335587 PMC3584223

[35] McRose DL, Baars O, Seyedsayamdost MR, Morel FMM. 2018. Quorum sensing and iron regulate a two-for-one siderophore gene cluster in *Vibrio harveyi*. Proc Natl Acad Sci USA 115:7581–7586. doi:10.1073/pnas.1805791115. 29954861 PMC6055174

[36] Di Lorenzo M, Poppelaars S, Stork M, Nagasawa M, Tolmasky ME, Crosa JH. 2004. A nonribosomal peptide synthetase with a novel domain organization is essential for siderophore biosynthesis in *Vibrio anguillarum*. J Bacteriol 186:7327–7336. doi:10.1128/JB.186.21.7327-7336.2004. 15489444 PMC523186

[37] Naka H, López CS, Crosa JH. 2010. Role of the pJM1 plasmid-encoded transport proteins FatB, C and D in ferric anguibactin uptake in the fish pathogen *Vibrio anguillarum*. Environ Microbiol Rep 2:104–111. doi:10.1111/j.1758-2229.2009.00110.x. 21304833 PMC3034151

[38] Tolmasky ME, Actis LA, Crosa JH. 1988. Genetic analysis of the iron uptake region of the *Vibrio anguillarum* plasmid pJM1: molecular cloning of genetic determinants encoding a novel trans activator of siderophore biosynthesis. J Bacteriol 170:1913–1919. doi:10.1128/jb.170.4.1913-1919.1988. 2832388 PMC211050

[39] Bagg A, Neilands JB. 1987. Ferric uptake regulation protein acts as a repressor, employing iron(II) as a cofactor to bind the operator of an iron transport operon in *Escherichia coli*. Biochemistry 26:5471–5477. doi:10.1021/bi00391a039. 2823881

[40] Wretlind B, Möllby R, Wadström T. 1971. Separation of two hemolysins from *Aeromonas hydrophila* by isoelectric focusing. Infect Immun 4:503–505. doi:10.1128/iai.4.4.503-505.1971. 5154892 PMC416338

[41] Bernheimer AW, Avigad LS, Avigad G. 1975. Interactions between aerolysin, erythrocytes, and erythrocyte membranes. Infect Immun 11:1312–1319. doi:10.1128/iai.11.6.1312-1319.1975. 166917 PMC415217

[42] Podobnik M, Kisovec M, Anderluh G. 2017. Molecular mechanism of pore formation by aerolysin-like proteins. Philos Trans R Soc Lond B Biol Sci 372:20160209. doi:10.1098/rstb.2016.0209. 28630149 PMC5483512

[43] Macpherson HL, Bergh Ø, Birkbeck TH. 2012. An aerolysin-like enterotoxin from *Vibrio splendidus* may be involved in intestinal tract damage and mortalities in turbot, *Scophthalmus maximus* (L.), and cod, *Gadus morhua* L., larvae. J Fish Dis 35:153–167. doi:10.1111/j.1365-2761.2011.01331.x. 22233514

[44] Rasmussen-Ivey CR, Figueras MJ, McGarey D, Liles MR. 2016. Virulence factors of *Aeromonas hydrophila*: in the wake of reclassification. Front Microbiol 7:1337. doi:10.3389/fmicb.2016.01337. 27610107 PMC4997093

[45] Mitrophanov AY, Groisman EA. 2008. Positive feedback in cellular control systems. Bioessays 30:542–555. doi:10.1002/bies.20769. 18478531 PMC2486260

[46] Rao SD, Igoshin OA. 2021. Overlaid positive and negative feedback loops shape dynamical properties of PhoPQ two-component system. PLoS Comput Biol 17:e1008130. doi:10.1371/journal.pcbi.1008130. 33395414 PMC7808668

[47] Mitarai N, Jensen MH, Semsey S. 2015. Coupled positive and negative feedbacks produce diverse gene expression patterns in colonies. mBio 6:e00059-15. doi:10.1128/mBio.00059-15. 25852158 PMC4453545

[48] Miller MB, Skorupski K, Lenz DH, Taylor RK, Bassler BL. 2002. Parallel quorum sensing systems converge to regulate virulence in *Vibrio cholerae*. Cell 110:303–314. doi:10.1016/s0092-8674(02)00829-2. 12176318

[49] Ruwandeepika HAD, Karunasagar I, Bossier P, Defoirdt T. 2015. Expression and quorum sensing regulation of type III secretion system genes of *Vibrio harveyi* during infection of gnotobiotic brine shrimp. PLoS One 10:e0143935. doi:10.1371/journal.pone.0143935. 26636765 PMC4670211

[50] Balado M, Lages MA, Fuentes-Monteverde JC, Martínez-Matamoros D, Rodríguez J, Jiménez C, Lemos ML. 2018. The siderophore piscibactin is a relevant virulence factor for *Vibrio anguillarum* favored at low temperatures. Front Microbiol 9:1766. doi:10.3389/fmicb.2018.01766. 30116232 PMC6083037

[51] Cuthbertson L, Nodwell JR. 2013. The TetR family of regulators. Microbiol Mol Biol Rev 77:440–475. doi:10.1128/MMBR.00018-13. 24006471 PMC3811609

[52] Bassler BL, Greenberg EP, Stevens AM. 1997. Cross-species induction of luminescence in the quorum-sensing bacterium *Vibrio harveyi*. J Bacteriol 179:4043–4045. doi:10.1128/jb.179.12.4043-4045.1997. 9190823 PMC179216

[53] Lin B, Wang Z, Malanoski AP, O’Grady EA, Wimpee CF, Vuddhakul V, Alves N, Thompson FL, Gomez-Gil B, Vora GJ. 2010. Comparative genomic analyses identify the *Vibrio harveyi* genome sequenced strains BAA-1116 and HY01 as *Vibrio campbellii*. Environ Microbiol Rep 2:81–89. doi:10.1111/j.1758-2229.2009.00100.x. 20686623 PMC2912166

[54] Boettcher KJ, Ruby EG. 1990. Depressed light emission by symbiotic *Vibrio fischeri* of the sepiolid squid *Euprymna scolopes*. J Bacteriol 172:3701–3706. doi:10.1128/jb.172.7.3701-3706.1990. 2163384 PMC213346

[55] Bassler BL, Wright M, Silverman MR. 1994. Multiple signalling systems controlling expression of luminescence in *Vibrio harveyi*: sequence and function of genes encoding a second sensory pathway. Mol Microbiol 13:273–286. doi:10.1111/j.1365-2958.1994.tb00422.x. 7984107

[56] Greenberg EP, Hastings JW, Ulitzur S. 1979. Induction of luciferase synthesis in *Beneckea harveyi* by other marine bacteria. Arch Microbiol 120:87–91. doi:10.1007/BF00409093.

[57] Gibson DG, Young L, Chuang R-Y, Venter JC, Hutchison CA, III, Smith HO. 2009. Enzymatic assembly of DNA molecules up to several hundred kilobases. Nat Methods 6:343–345. doi:10.1038/nmeth.1318. 19363495

[58] Edwards RA, Keller LH, Schifferli DM. 1998. Improved allelic exchange vectors and their use to analyze 987P fimbria gene expression. Gene 207:149–157. doi:10.1016/s0378-1119(97)00619-7. 9511756

[59] Chaparian RR, Olney SG, Hustmyer CM, Rowe-Magnus DA, van Kessel JC. 2016. Integration host factor and LuxR synergistically bind DNA to coactivate quorum-sensing genes in *Vibrio harveyi*. Mol Microbiol 101:823–840. doi:10.1111/mmi.13425. 27191515

[60] Mukherjee S, Moustafa D, Smith CD, Goldberg JB, Bassler BL. 2017. The RhlR quorum-sensing receptor controls *Pseudomonas aeruginosa* pathogenesis and biofilm development independently of its canonical homoserine lactone autoinducer. PLoS Pathog 13:e1006504. doi:10.1371/journal.ppat.1006504. 28715477 PMC5531660

[61] Kim D, Pertea G, Trapnell C, Pimentel H, Kelley R, Salzberg SL. 2013. TopHat2: accurate alignment of transcriptomes in the presence of insertions, deletions and gene fusions. Genome Biol 14:R36. doi:10.1186/gb-2013-14-4-r36. 23618408 PMC4053844

[62] Love MI, Huber W, Anders S. 2014. Moderated estimation of fold change and dispersion for RNA-seq data with DESeq2. Genome Biol 15:550. doi:10.1186/s13059-014-0550-8. 25516281 PMC4302049

[63] Benson DA, Karsch-Mizrachi I, Lipman DJ, Ostell J, Rapp BA, Wheeler DL. 2000. GenBank. Nucleic Acids Res 28:15–18. doi:10.1093/nar/28.1.15. 10592170 PMC102453

[64] Smith TF, Waterman MS. 1981. Identification of common molecular subsequences. J Mol Biol 147:195–197. doi:10.1016/0022-2836(81)90087-5. 7265238

