# Supplementary figures and images for: LuxT Is a Global Regulator of Low-Cell-Density Behaviors, Including Type III Secretion, Siderophore Production, and Aerolysin Production, in Vibrio harveyi
Source: mBio. 2022 Jan 18;13(1):e03621-21. doi: 10.1128/mbio.03621-21 (PMC8764538; doi:10.1128/mbio.03621-21)

A

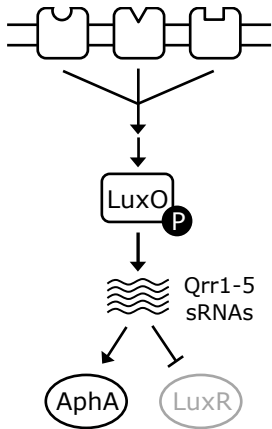

B

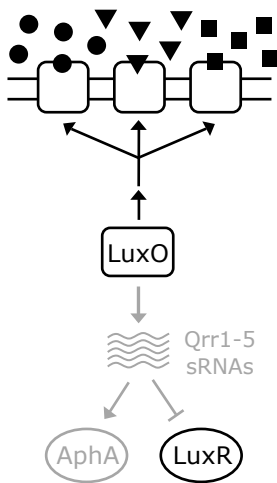

Supplement: FIG S1 [file mbio.03621-21-sf001.pdf]

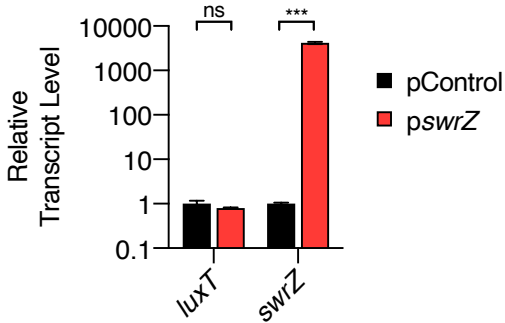

Supplement: FIG S2 [file mbio.03621-21-sf002.pdf]

A

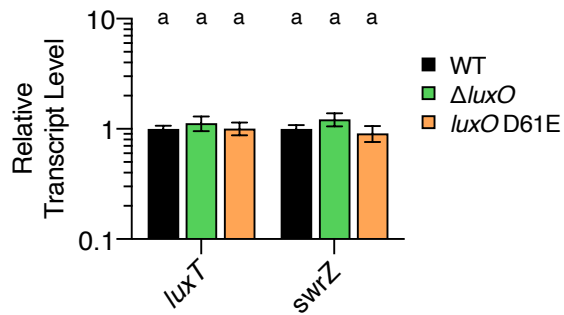

B

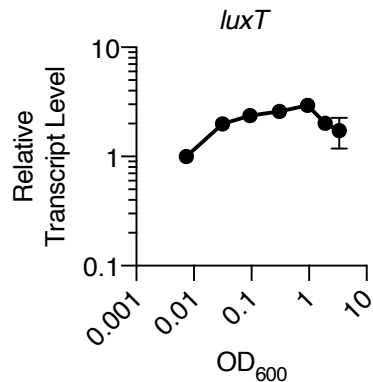

C

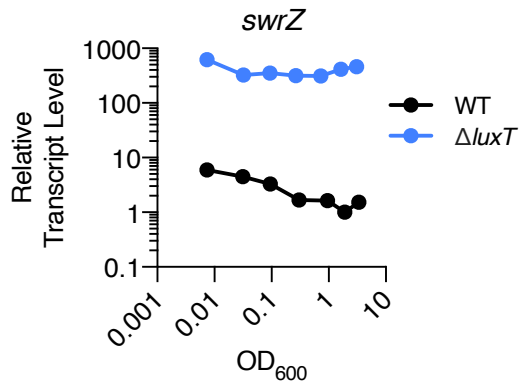

Supplement: FIG S3 [file mbio.03621-21-sf003.pdf]

A

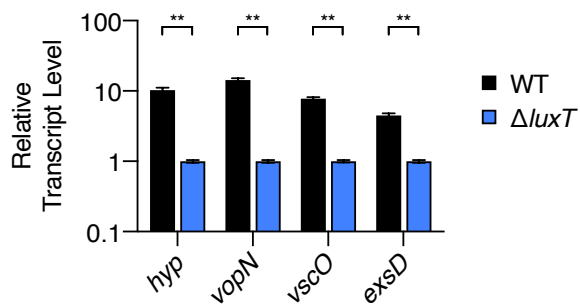

B

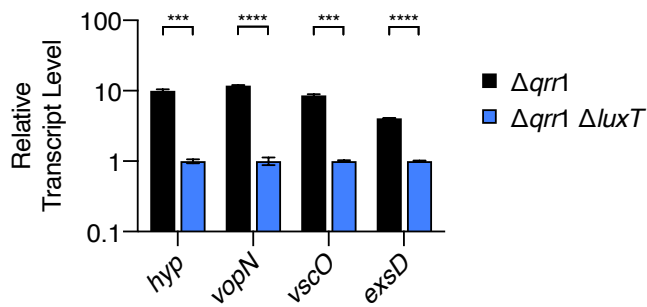

C

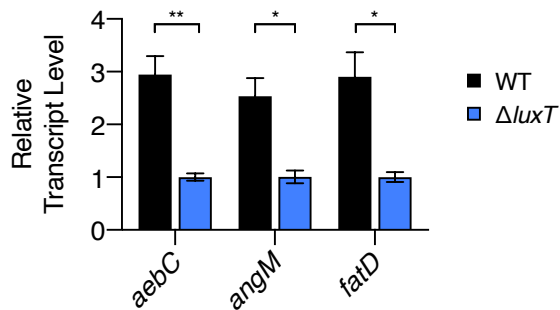

D

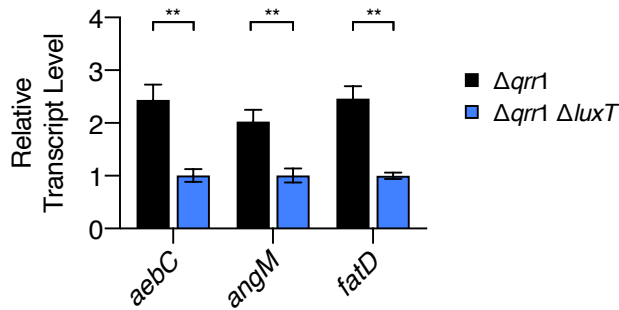

E

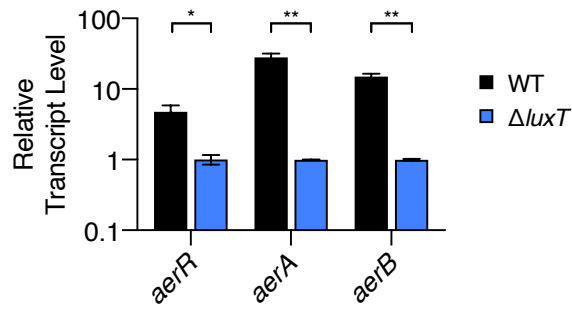

F

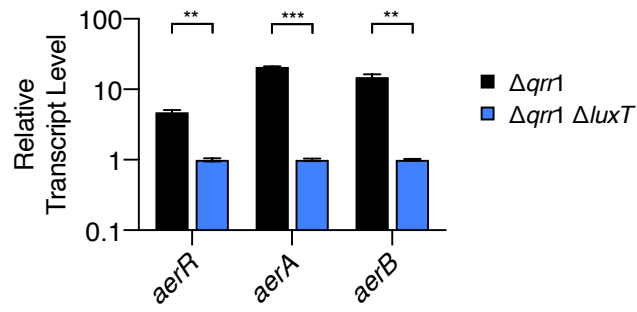

Supplement: FIG S4 [file mbio.03621-21-sf004.pdf]

**A**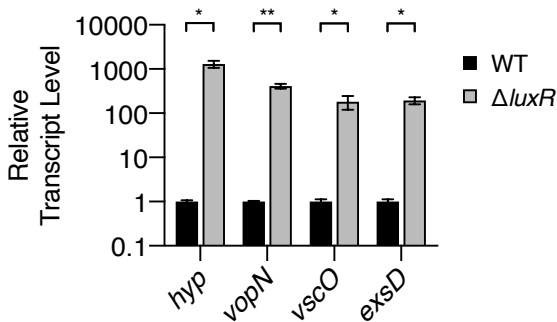**B**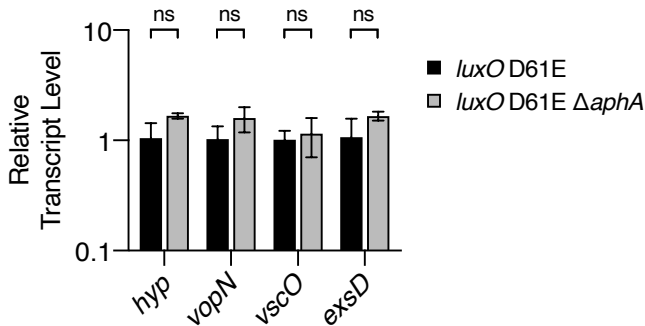

Supplement: FIG S5 [file mbio.03621-21-sf005.pdf]

A

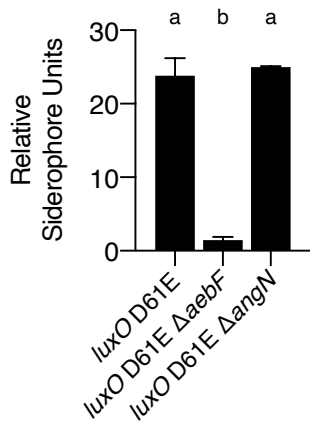

B

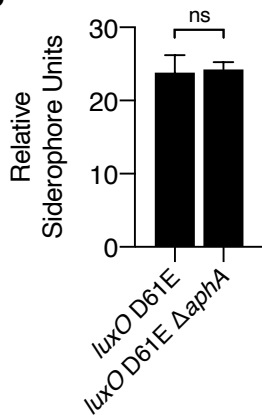

C

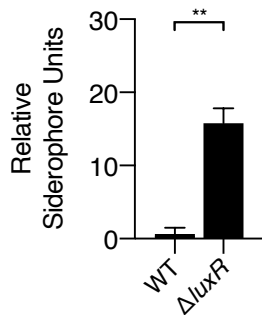

D

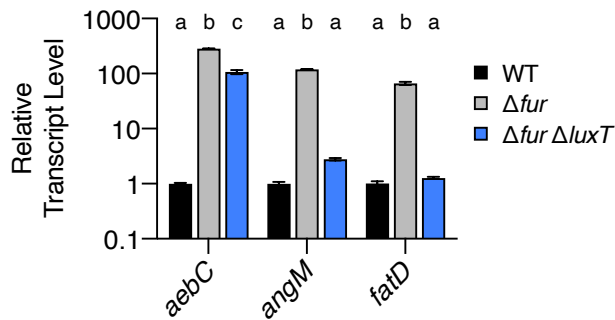

E

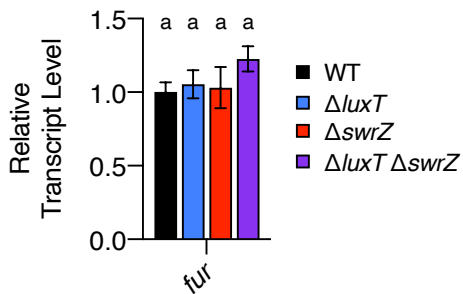

Supplement: FIG S6 [file mbio.03621-21-sf006.pdf]

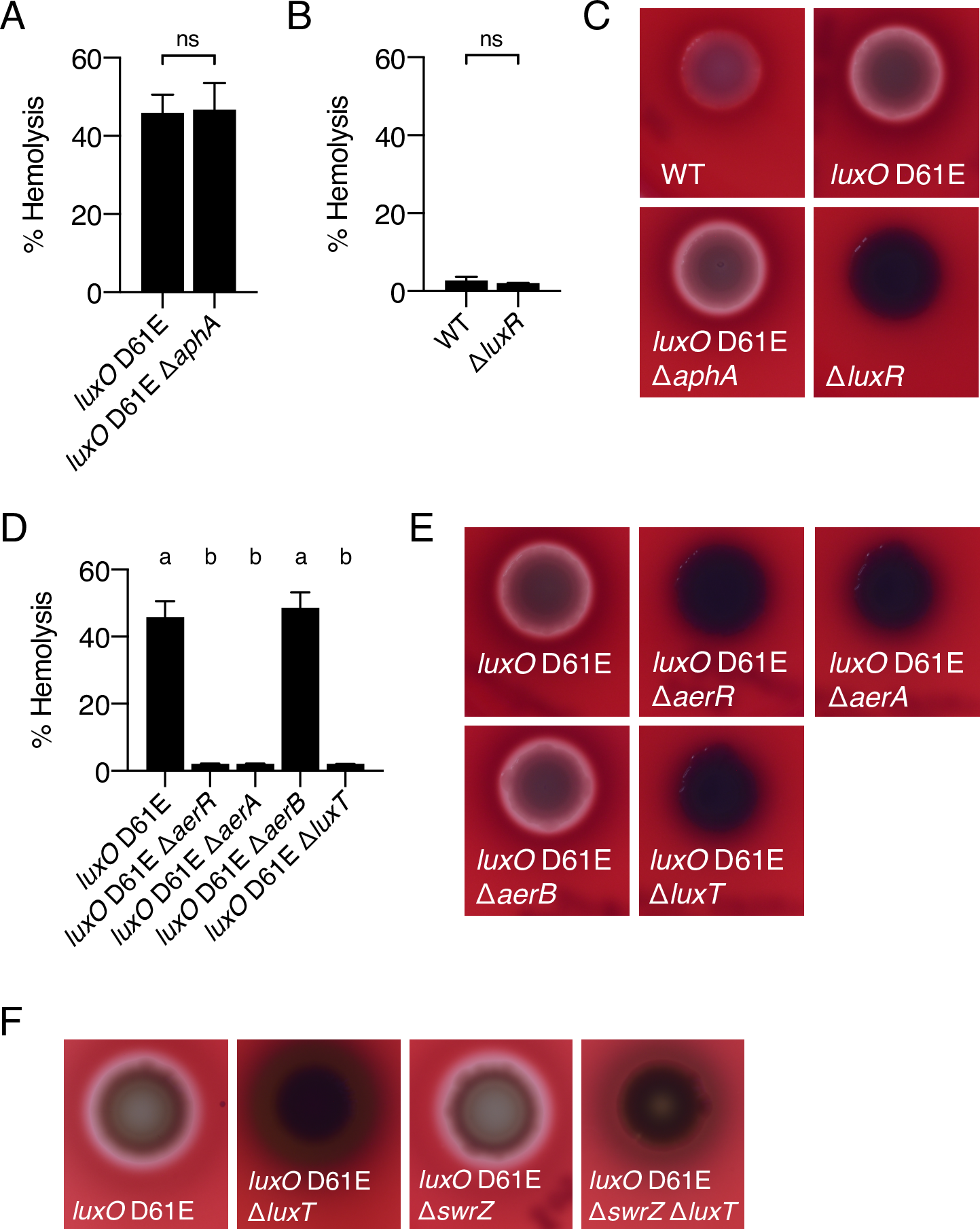

Supplement: FIG S7 [file mbio.03621-21-sf007.tif]

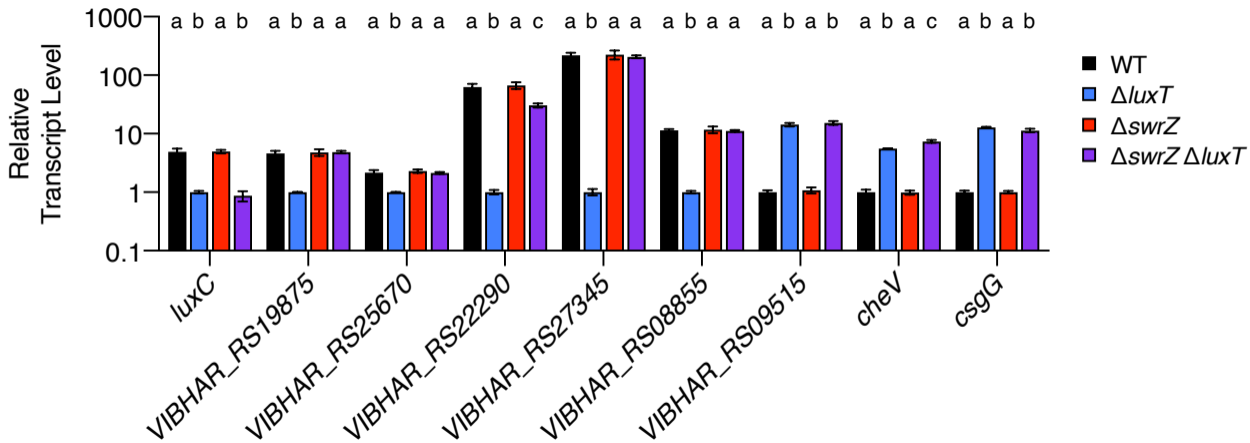

Supplement: FIG S8 [file mbio.03621-21-sf008.pdf]
