# Supplementary material for: LuxT Is a Global Regulator of Low-Cell-Density Behaviors, Including Type III Secretion, Siderophore Production, and Aerolysin Production, in Vibrio harveyi
Source: mBio. 2022 Jan 18;13(1):e03621-21. doi: 10.1128/mbio.03621-21 (PMC8764538; doi:10.1128/mbio.03621-21)
Supplement: TABLE S1 [file mbio.03621-21-st001.docx]

**Table S1**

**A. Strains used in this study**

(WT strains are depicted in bold with variants listed below them.)

| **Strain** | **Relevant genotype or feature** | **Reference** |
| --- | --- | --- |
|  |  |  |
| ***V. harveyi* BB120** | WT | (1) |
| ME287 | Δ*luxT* | (2) |
| ME291 | Δ*swrZ* | (2) |
| ME1057 | Δ*luxA* | This study |
| ME1056 | Δ*luxA* Δ*luxT* | This study |
| BB721 | Δ*luxO* | (3) |
| JSV780 | *luxO* D61E | (4) |
| KT39 | Δ*qrr*1 | (5) |
| ME431 | Δ*qrr*1 Δ*luxT* | (2) |
| KM669 | Δ*luxR* | (6) |
| STR416 | *luxO* D61E Δ*aphA* | (7) |
| ME302 | Δ*swrZ* Δ*luxT* | (2) |
| ME181 | *luxO* D61E Δ*luxT* | (2) |
| ME805 | *luxO* D61E Δ*swrZ* | This study |
| ME807 | *luxO* D61E Δ*swrZ* Δ*luxT* | This study |
| ME944 | Δ*luxR* Δ*swrZ* Δ*luxT* | This study |
| ME942 | Δ*luxR* Δ*swrZ* | This study |
| ME1044 | Δ*exsA* | This study |
| ME1032 | Δ*exsA* Δ*luxT* | This study |
| ME132 | *luxO* D61E Δ*aebF* | This study |
| ME133 | *luxO* D61E Δ*angN* | This study |
| ME1009 | Δ*fur* | This study |
| ME1010 | Δ*fur* Δ*luxT* | This study |
| ME1042 | *luxO* D61E Δ*aerR* | This study |
| ME1019 | *luxO* D61E Δ*aerA* | This study |
| ME1017 | *luxO* D61E Δ*aerB* | This study |
| ***A. fischeri* ES114** | WT | (8) |
| ME226 | Δ*luxT* | (9) |
| ME1030 | Δ*swrZ* | This study |
| ME1050 | Δ*swrZ* Δ*luxT* | This study |
| ***E. coli* S17-1 λ*pir*** | WT | (10) |
| ***E. coli* MG1655** | WT | (11) |
|  |  |  |

**B. Primers used in this study**

Sequences are provided in the 5’ → 3’ direction

| **Name** | **Sequence** | **Description** |
| --- | --- | --- |
|  |  |  |
| STR-0040 | CGTGAGCGTATCCCGGTATCTAT | qRT-PCR, *hfq* (*V. harveyi)* |
| STR-0041 | TTGCAGTTTGATACCGTTCACAAG | qRT-PCR, *hfq* (*V. harveyi)* |
| ME-405 | TGAAACAGAAATACAACGTTGGTGC | qRT-PCR, *swrZ* (*V. harveyi)* |
| ME-501 | CACAGGTGCAACCCAACAGC | qRT-PCR, *swrZ* (*V. harveyi)* |
| ME-605 | TCTAGAGGCATCAAATAAAACGAAAG | Plasmid construction, pSC101Kan |
| ME-606 | AAGACGGGTAAGCCTGTTGATG | Plasmid construction, pSC101Kan |
| ME-607 | AGCGGTATCATCAACAGGCTTACCCGTCTTCCTGTTAAGTATCTTCCTGGCATC | Plasmid construction, pSC101Kan |
| ME-608 | GAGCCTTTCGTTTTATTTGATGCCTCTAGACACATGGTCCTTCTTGAGTTTGTAAC | Plasmid construction, pSC101Kan |
| ME-611 | ACATGGCATGGATGAACTGTACAAC | Plasmid construction, P*_swrZ_*-*lux* and P*_luxT_*-*lux* (pSC101Kan vector) |
| ME-612 | GTTACAAACTCAAGAAGGACCATGTG | Plasmid construction, P*_swrZ_*-*lux* and P*_luxT_*-*lux* (pSC101Kan vector) |
| ME-1121 | TAGGAATTCAATTAGGAGGTAATTAAGCATGGAAAAACACTTACCTTTAATAATAAATGG | Plasmid construction, P*_swrZ_*-*lux* and P*_luxT_*-*lux* (*luxCDABE*) |
| ME-1122 | TAGACACATGGTCCTTCTTGAGTTTGTAACTTATTACAAATAAGCGAACGCGTCC | Plasmid construction, P*_swrZ_*-*lux* and P*_luxT_*-*lux* (*luxCDABE*) |
| ME-714 | CCTCAGTTGTACAGTTCATCCATGCCATGT AAAAAGAGGGCTTCAGCCCTC | Plasmid construction, P*_swrZ_*-*lux* (P*_swrZ_*) |
| ME-604 | CCTAGGCCTGTCGAGGCTGTTTCCTGTGTG ATTATCGTGACGACAACTAAACAGTAC | Plasmid construction, P*_swrZ_*-*lux* (P*_swrZ_*) |
| ME-1032 | GTTTGATCTGCTTAATAAATCTGGTTAAAA | P*_luxC_* Control EMSA probe forward primer |
| ME-1033 | TCCATATCAAGAGCTTCTCCTTTG | P*_luxC_* Control EMSA probe reverse primer |
| ME-1190 | AATCATCAGTTCAATAAAAAAGAGGGC | P*_swrZ_* EMSA probe forward primer |
| ME-1174 | TAAACAGTACGTAGTTCGGTTGTAATTG | P*_swrZ_* EMSA probe reverse primer |
| ME-443 | GCTTAATTACCTCCTCTTCCTTAGCTCCTGAATTCCTAG | Plasmid construction, p*swrZ* (pFED343 vector) |
| ME-444 | TCACTACTCTGTGCTATGGTGTTC | Plasmid construction, p*swrZ* (pFED343 vector) |
| ME-839 | CAGGAGCTAAGGAAGAGGAGGTAATTAAGCATGAGGTTGCTTGTGTCTAGTCC | Plasmid construction, p*swrZ* (*swrZ*) |
| ME-840 | AGCATTGAACACCATAGCACAGAGTAGTGATTAAGCAGGCTCTAGCTCTAGTAC | Plasmid construction, p*swrZ* (*swrZ*) |
| ME-128 | GAAGATCATGGATGCCGTTGTTG | qRT-PCR, *luxT* (*V. harveyi)* |
| ME-415 | AATGGTGGCTAATACCTGTACGC | qRT-PCR, *luxT* (*V. harveyi)* |
| ME-1130 | CCTCAGTTGTACAGTTCATCCATGCCATGTCGAATCCTCTTGTAAATACTGTGGG | Plasmid construction, P*_luxT_*-*lux* (P*_luxT_*) |
| ME-1131 | CCTAGGCCTGTCGAGGCTGTTTCCTGTGTGTTATGGCATATTTTAAGCTCTTCTCTTTG | Plasmid construction, P*_luxT_*-*lux* (P*_luxT_*) |
| pRE112-F | ATGCAGTTCACTTACACCGCTTC | Plasmid construction, pRE112 mediated chromosomal alterations |
| pRE112-R | GGGATCGGGCCCTATCACTT | Plasmid construction, pRE112 mediated chromosomal alterations |
| ME-1191 | GGGTTGAGAAGCGGTGTAAGTGAACTGCATGTCGGTGGTGCACATGATGC | Plasmid construction, *luxA* deletion (*V. harveyi)* |
| ME-1192 | AGGTGGCTGATAAGTGAGAAGG | Plasmid construction, *luxA* deletion (*V. harveyi)* |
| ME-1193 | GGAAACTTCCTTCTCACTTATCAGCCACCTATTATCGCATCTATGAAGCTATTCC | Plasmid construction, *luxA* deletion (*V. harveyi)* |
| ME-1194 | ACGCCTGAATAAGTGATAGGGCCCGATCCCTGCGCTTTATCTTCCATCGAC | Plasmid construction, *luxA* deletion (*V. harveyi)* |
| ME-1172 | CAAGGACATACAATACATAATGCTGAG | P*_luxT_* EMSA probe forward primer |
| ME-1173 | TGGCATATTTTAAGCTCTTCTCTTTG | P*_luxT_* EMSA probe reverse primer |
| ME-1184 | GGAGTTCTCAATACAACAGCCAATC | qRT-PCR, *hyp* (*V. harveyi)* |
| ME-1185 | GATTCGGGTCGATATGGGCATC | qRT-PCR, *hyp* (*V. harveyi)* |
| ME-817 | TCCGCGTACACAATGCAACTC | qRT-PCR, *vopN* (*V. harveyi)* |
| ME-818 | CGTCTTTCACTTTGCGTTTCG | qRT-PCR, *vopN* (*V. harveyi)* |
| ME-506 | GGTTCAGCAACAAGAGTATCGAG | qRT-PCR, *vscO* (*V. harveyi)* |
| ME-507 | CGCCGTTCTTCTTCCTCTTC | qRT-PCR, *vscO* (*V. harveyi)* |
| ME-1186 | TAACCCAATCATCGCAAGTGCC | qRT-PCR, *exsD* (*V. harveyi)* |
| ME-1187 | CGTTGCAGCAAAGACAATTGTTC | qRT-PCR, *exsD* (*V. harveyi)* |
| ME-1175 | CAGTCTCACATTGTTGTCGTTCAC | qRT-PCR, *exsA* (*V. harveyi)* |
| ME-1176 | CTTGAGAGAGGAACACACCACAAC | qRT-PCR, *exsA* (*V. harveyi)* |
| ME-1177 | GGGTTGAGAAGCGGTGTAAGTGAACTGCATATAAGTAAATAGAGTACGCAGTGAAATC | Plasmid construction, *exsA* deletion (*V. harveyi)* |
| ME-1178 | CACATCCATTTTCTACCCTTCATAATCC | Plasmid construction, *exsA* deletion (*V. harveyi)* |
| ME-1179 | TAGGATTATGAAGGGTAGAAAATGGATGTGGTCGCTATTGCTAAGTGAAATGTC | Plasmid construction, *exsA* deletion (*V. harveyi)* |
| ME-1180 | ACGCCTGAATAAGTGATAGGGCCCGATCCCCGCATTTAAGTCGCTATACACATG | Plasmid construction, *exsA* deletion (*V. harveyi)* |
| ME-823 | TTACCCAAGGCATTACCGCAG | qRT-PCR, *aebC* (*V. harveyi)* |
| ME-824 | TTTGGTTTCCCGCATCCTTTGC | qRT-PCR, *aebC* (*V. harveyi)* |
| ME-510 | GAACCAGAAAGCATCTCATGGC | qRT-PCR, *angM* (*V. harveyi)* |
| ME-511 | CATAGCGTAAATTGAGCCCATAATC | qRT-PCR, *angM* (*V. harveyi)* |
| ME-805 | GGTCACTACTGCTGAAGATGGAC | qRT-PCR, *fatD* (*V. harveyi)* |
| ME-806 | AGCCGGCCCCTGTTAGAATC | qRT-PCR, *fatD* (*V. harveyi)* |
| ME-10 | GGGTTGAGAAGCGGTGTAAGTGAACTGCATATGTACTGCCGAAAACTCCCGTC | Plasmid construction, *aebF* deletion (*V. harveyi)* |
| ME-11 | TCCTTTGTTTTTCTTATTCTTTGGCTG | Plasmid construction, *aebF* deletion (*V. harveyi)* |
| ME-12 | TAGCAGCCAAAGAATAAGAAAAACAAAGGATTCGTCAACACAATCTAGGTCGAAC | Plasmid construction, *aebF* deletion (*V. harveyi)* |
| ME-13 | ACGCCTGAATAAGTGATAGGGCCCGATCCCAATCTCTCATTTCATGGACACTACTG | Plasmid construction, *aebF* deletion (*V. harveyi)* |
| ME-20 | GGGTTGAGAAGCGGTGTAAGTGAACTGCATCTTGCGCGATGGGATGTTAAG | Plasmid construction, *angN* deletion (*V. harveyi)* |
| ME-21 | CTGCTTTCACTTTGTTGACAATCAATC | Plasmid construction, *angN* deletion (*V. harveyi)* |
| ME-22 | ACCGATTGATTGTCAACAAAGTGAAAGCAGCACATCATTGATTTAGTAGCATATTTTC | Plasmid construction, *angN* deletion (*V. harveyi)* |
| ME-23 | ACGCCTGAATAAGTGATAGGGCCCGATCCC CCTCCTATCGAACGCTCATAAAG | Plasmid construction, *angN* deletion (*V. harveyi)* |
| ME-60 | GGGTTGAGAAGCGGTGTAAGTGAACTGCATGACCAAGAAGATTACGCAGAGTAC | Plasmid construction, *fur* deletion (*V. harveyi)* |
| ME-61 | ATACTTTCCCGTTGGATCATCTGC | Plasmid construction, *fur* deletion (*V. harveyi)* |
| ME-62 | ATCGCTGCAGATGATCCAACGGGAAAGTATAAAGTAACGCTTTTTGTTAAGAAAGAC | Plasmid construction, *fur* deletion (*V. harveyi)* |
| ME-63 | ACGCCTGAATAAGTGATAGGGCCCGATCCCCCGTAAAGTGAAAGGCGTAATTCAC | Plasmid construction, *fur* deletion (*V. harveyi)* |
| ME-1128 | GCCAGACTGCCAGCACATC | qRT-PCR, *fur* (*V. harveyi)* |
| ME-1129 | CATCATCGAATTGGTTTAGTACACG | qRT-PCR, *fur* (*V. harveyi)* |
| ME-813 | TCTTACCCTTTTACTGGAACGCTC | qRT-PCR, *aerR* (*V. harveyi)* |
| ME-814 | GCTAGCGAAGTTTCACCGATG | qRT-PCR, *aerR* (*V. harveyi)* |
| ME-512 | CCTATCTACATTCGCAACTGGC | qRT-PCR, *aerA* (*V. harveyi)* |
| ME-513 | TCAAGTGGACGATAGCCTGAAC | qRT-PCR, *aerA* (*V. harveyi)* |
| ME-782 | GGCTATCATGGGGAGATCAAGTC | qRT-PCR, *aerB* (*V. harveyi)* |
| ME-783 | GGTGATGGGCATTGAGACGTTAC | qRT-PCR, *aerB* (*V. harveyi)* |
| ME-1198 | GGGTTGAGAAGCGGTGTAAGTGAACTGCATAACGGGTGGTTTCTTGGGTGC | Plasmid construction, *aerR* deletion (*V. harveyi)* |
| ME-1199 | TTTAATAGGGTTATATAAGTTCATACGACAG | Plasmid construction, *aerR* deletion (*V. harveyi)* |
| ME-1200 | TGTCGTATGAACTTATATAACCCTATTAAATGAAACCCTATATCGCTGTTGCC | Plasmid construction, *aerR* deletion (*V. harveyi)* |
| ME-1201 | ACGCCTGAATAAGTGATAGGGCCCGATCCCGCCAGTTGCGAATGTAGATAGG | Plasmid construction, *aerR* deletion (*V. harveyi)* |
| ME-1153 | GGGTTGAGAAGCGGTGTAAGTGAACTGCATACACCAGACCAGTTTGAGCAAG | Plasmid construction, *aerA* deletion (*V. harveyi)* |
| ME-1154 | AAGGCTGATGTTGATGTTTCGC | Plasmid construction, *aerA* deletion (*V. harveyi)* |
| ME-1155 | ACGTTCATGCGAAACATCAACATCAGCCTTGCTCTTGGTTTTGAAGGTGCTC | Plasmid construction, *aerA* deletion (*V. harveyi)* |
| ME-1156 | ACGCCTGAATAAGTGATAGGGCCCGATCCCGAGGGTTAGCCTGATGAGAGTG | Plasmid construction, *aerA* deletion (*V. harveyi)* |
| ME-1141 | GGGTTGAGAAGCGGTGTAAGTGAACTGCATAGCATACAAGCAGCAGACTAAG | Plasmid construction, *aerB* deletion (*V. harveyi)* |
| ME-1142 | CATACTGAGCTGCGTACTGAC | Plasmid construction, *aerB* deletion (*V. harveyi)* |
| ME-1143 | ATGCCTAACGTCAGTACGCAGCTCAGTATGCGCCTACTCATTCAACCTCTAG | Plasmid construction, *aerB* deletion (*V. harveyi)* |
| ME-1144 | ACGCCTGAATAAGTGATAGGGCCCGATCCCCGCATTGATGAAGTCAAGCAG | Plasmid construction, *aerB* deletion (*V. harveyi)* |
| ME-416 | TATACAACAGGGCAGCGTTGG | qRT-PCR, *luxC* (*V. harveyi)* |
| ME-417 | TCCAATTTGCTTCGAGTTTCGC | qRT-PCR, *luxC* (*V. harveyi)* |
| ME-809 | TGCTAAGCGAATACTTTGAAGGAC | qRT-PCR, *VIBHAR_RS19875* (*V. harveyi)* |
| ME-810 | AAGAACGAGATCTGGCTGGC | qRT-PCR, *VIBHAR_RS19875* (*V. harveyi)* |
| ME-780 | AGACAACAGCTCCAATACGGC | qRT-PCR, *VIBHAR_RS25670* (*V. harveyi)* |
| ME-781 | ATTTGGGTTGGCTTTGGTCTCTAC | qRT-PCR, *VIBHAR_RS25670* (*V. harveyi)* |
| ME-1118 | AGTAATCCAAGCGGCTCAAACC | qRT-PCR, *VIBHAR_RS22290* (*V. harveyi)* |
| ME-1119 | TTTTCCATGATACTCGACCAGTCAC | qRT-PCR, *VIBHAR_RS22290* (*V. harveyi)* |
| ME-516 | CGCTGGTAATGGTAATTCCACTAG | qRT-PCR, *VIBHAR_RS27345* (*V. harveyi)* |
| ME-517 | TAGATCGACAAACACTAAACCTCTG | qRT-PCR, *VIBHAR_RS27345* (*V. harveyi)* |
| ME-514 | TACAACAAGCACGCAAAGCG | qRT-PCR, *VIBHAR_RS08855* (*V. harveyi)* |
| ME-515 | ATCTGCACGGTTACGTTCTGG | qRT-PCR, *VIBHAR_RS08855* (*V. harveyi)* |
| ME-831 | CCACGCAACAAACGATGCAG | qRT-PCR, *VIBHAR_RS09515* (*V. harveyi)* |
| ME-832 | TCTCTCTCAAATCACAGTTCAACAC | qRT-PCR, *VIBHAR_RS09515* (*V. harveyi)* |
| ME-835 | TACAGTGCCCTAAGCTGACTTC | qRT-PCR, *cheV* (*V. harveyi)* |
| ME-836 | CAATATCTGTGGTTGGACGGCC | qRT-PCR, *cheV* (*V. harveyi)* |
| ME-502 | GTAGCCATTCACAAGCTGAGC | qRT-PCR, *csgG* (*V. harveyi)* |
| ME-503 | ATTCGACCTAATGCGACTTTGC | qRT-PCR, *csgG* (*V. harveyi)* |
| ME-93 | GTTAACGGGATCAAACTACAGGGAC | qRT-PCR, *hfq* (*A. fischeri)* |
| ME-94 | AGTAGAAATCGCATGCTTGTATACC | qRT-PCR, *hfq* (*A. fischeri)* |
| ME-1239 | TGATCCACCAAGATATTCTCTCTGG | qRT-PCR, *swrZ* (*A. fischeri)* |
| ME-1240 | AGCTTCACGAATAGGGGATGC | qRT-PCR, *swrZ* (*A. fischeri)* |
| ME-1223 | GGGTTGAGAAGCGGTGTAAGTGAACTGCATCGTTTCTGTTCAACTTCCAGCTC | Plasmid construction, *swrZ* deletion (*A. fischeri)* |
| ME-1224 | TTTTCCAGAGAGAATATCTTGGTGG | Plasmid construction, *swrZ* deletion (*A. fischeri)* |
| ME-1225 | ATGATCCACCAAGATATTCTCTCTGGAAAATACTCGAAAGCCAGTGATCTTATATC | Plasmid construction, *swrZ* deletion (*A. fischeri)* |
| ME-1226 | ACGCCTGAATAAGTGATAGGGCCCGATCCCCTTTCGATGTATTGCTATCTGAGAG | Plasmid construction, *swrZ* deletion (*A. fischeri)* |
|  |  |  |

**C. Plasmids used in this study**

| **Plasmid Name** | **Stock Name** | **Description** | **Origin, marker** | **Reference** |
| --- | --- | --- | --- | --- |
|  |  |  |  |  |
| pSC101Kan | pME94 | Low-copy vector. pSC101 origin from pJES-24 (12) and Kan^R^ cassette from pEVS143 (13) | pSC101,  Kan^R^ | This study |
| P*_swrZ_*-*lux* | pME161 | *swrZ-luxCDABE* transcriptional reporter in pME94 | pSC101,  Kan^R^ | This study |
| pKP8-35 | pKP8-35 | P*_BAD_* overexpression vector | pBR322,  Amp^R^ | (14) |
| p*luxT* | pME109 | *V. harveyi luxT* overexpression vector, cloned in pKP8-35 | pBR322,  Amp^R^ | (2) |
| pFED343 | pFED343 | P*_tac_* overexpression vector | P15A, Cam^R^ | (15) |
| p*swrZ* | pME192 | *V. harveyi swrZ* overexpression vector, cloned in pFED343 | P15A, Cam^R^ | This study |
| P*_luxT_*-*lux* | pME186 | *luxT-luxCDABE* transcriptional reporter in pME94 | pSC101,  Kan^R^ | This study |
| pRE112 | pRE112 | allelic exchange vector harboring *sacB* as a counter-selectable marker | R6Kγ, Cam^R^ | (16) |
| pRE112-Δ*luxA* | pME213 | *V. harveyi luxA* deletion construct in pRE112 | R6Kγ, Cam^R^ | This study |
| pRE112-Δ*swrZ* | pME64 | *V. harveyi swrZ* deletion construct in pRE112 | R6Kγ, Cam^R^ | (2) |
| p*luxT* | pME69 | *V. harveyi luxT* overexpression vector, cloned in pFED343 | P15A, Cam^R^ | (2) |
| pRE112-Δ*luxT* | pME12 | *V. harveyi luxT* deletion construct in pRE112 | R6Kγ, Cam^R^ | (2) |
| pRE112-Δ*exsA* | pME212 | *V. harveyi exsA* deletion construct in pRE112 | R6Kγ, Cam^R^ | This study |
| pRE112-Δ*aebF* | pME1 | *V. harveyi aebF* deletion construct in pRE112 | R6Kγ, Cam^R^ | This study |
| pRE112-Δ*angN* | pME3 | *V. harveyi angN* deletion construct in pRE112 | R6Kγ, Cam^R^ | This study |
| pRE112-Δ*fur* | pME9 | *V. harveyi fur* deletion construct in pRE112 | R6Kγ, Cam^R^ | This study |
| pRE112-Δ*aerR* | pME219 | *V. harveyi aerR* deletion construct in pRE112 | R6Kγ, Cam^R^ | This study |
| pRE112-Δ*aerA* | pME215 | *V. harveyi aerA* deletion construct in pRE112 | R6Kγ, Cam^R^ | This study |
| pRE112-Δ*aerB* | pME214 | *V. harveyi aerB* deletion construct in pRE112 | R6Kγ, Cam^R^ | This study |
| pRE112-Δ*swrZ* (*A. fischeri*) | pME220 | *A. fischeri swrZ* deletion construct in pRE112 | R6Kγ, Cam^R^ | This study |
|  |  |  |  |  |

**Table S1 References**

1. Bassler BL, Greenberg EP, Stevens AM. 1997. Cross-species induction of luminescence in the quorum-sensing bacterium *Vibrio harveyi*. J Bacteriol 179:4043–4045.

2. Eickhoff MJ, Fei C, Huang X, Bassler BL. 2021. LuxT controls specific quorum-sensing-regulated behaviors in *Vibrionaceae* spp. via repression of *qrr*1, encoding a small regulatory RNA. PLoS Genet 17:e1009336.

3. Bassler BL, Wright M, Silverman MR. 1994. Sequence and function of LuxO, a negative regulator of luminescence in *Vibrio harveyi*. Mol Microbiol 12:403–412.

4. Freeman JA, Bassler BL. 1999. A genetic analysis of the function of LuxO, a two-component response regulator involved in quorum sensing in *Vibrio harveyi*. Mol Microbiol 31:665–677.

5. Tu KC, Bassler BL. 2007. Multiple small RNAs act additively to integrate sensory information and control quorum sensing in *Vibrio harveyi*. Genes Dev 21:221–233.

6. Pompeani AJ, Irgon JJ, Berger MF, Bulyk ML, Wingreen NS, Bassler BL. 2008. The *Vibrio harveyi* master quorum-sensing regulator, LuxR, a TetR-type protein is both an activator and a repressor: DNA recognition and binding specificity at target promoters. Mol Microbiol 70:76–88.

7. van Kessel JC, Rutherford ST, Shao Y, Utria AF, Bassler BL. 2013. Individual and combined roles of the master regulators AphA and LuxR in control of the *Vibrio harveyi* quorum-sensing regulon. J Bacteriol 195:436–443.

8. Boettcher KJ, Ruby EG. 1990. Depressed light emission by symbiotic *Vibrio fischeri* of the sepiolid squid *Euprymna scolopes*. J Bacteriol 172:3701–3706.

9. Eickhoff MJ, Bassler BL. 2020. *Vibrio fischeri* siderophore production drives competitive exclusion during dual-species growth. Mol Microbiol 114:244–261.

10. de Lorenzo V, Timmis KN. 1994. Analysis and construction of stable phenotypes in gram-negative bacteria with Tn*5*- and Tn*10*-derived minitransposons. Methods Enzymol 235:386–405.

11. Blattner FR, Plunkett G, Bloch CA, Perna NT, Burland V, Riley M, Collado-Vides J, Glasner JD, Rode CK, Mayhew GF, Gregor J, Davis NW, Kirkpatrick HA, Goeden MA, Rose DJ, Mau B, Shao Y. 1997. The complete genome sequence of *Escherichia coli* K-12. Science 277:1453–1462.

12. Papenfort K, Silpe JE, Schramma KR, Cong J-P, Seyedsayamdost MR, Bassler BL. 2017. A *Vibrio cholerae* autoinducer-receptor pair that controls biofilm formation. Nat Chem Biol 13:551–557.

13. Dunn AK, Millikan DS, Adin DM, Bose JL, Stabb EV. 2006. New rfp- and pES213-derived tools for analyzing symbiotic *Vibrio fischeri* reveal patterns of infection and *lux* expression in situ. Appl Environ Microbiol 72:802–810.

14. Papenfort K, Pfeiffer V, Mika F, Lucchini S, Hinton JCD, Vogel J. 2006. SigmaE-dependent small RNAs of *Salmonella* respond to membrane stress by accelerating global *omp* mRNA decay. Mol Microbiol 62:1674–1688.

15. Swem LR, Swem DL, Wingreen NS, Bassler BL. 2008. Deducing receptor signaling parameters from in vivo analysis: LuxN/AI-1 quorum sensing in *Vibrio harveyi*. Cell 134:461–473.

16. Edwards RA, Keller LH, Schifferli DM. 1998. Improved allelic exchange vectors and their use to analyze 987P fimbria gene expression. Gene 207:149–157.
